# RNA-binding is an ancient trait of the Annexin family

**DOI:** 10.3389/fcell.2023.1161588

**Published:** 2023-06-15

**Authors:** Sudarshan S. Patil, Vipul Panchal, Trude Røstbø, Sofya Romanyuk, Hanne Hollås, Ruth Brenk, Ann Kari Grindheim, Anni Vedeler

**Affiliations:** ^1^ Neurotargeting Group, Department of Biomedicine, University of Bergen, Bergen, Norway; ^2^ Biorecognition Unit, Department of Biomedicine, University of Bergen, Bergen, Norway

**Keywords:** Annexin, RNA-binding, mRNP complexes, mRNA, RNA granules, c-Myc, 3′untranslated region

## Abstract

**Introduction:** The regulation of intracellular functions in mammalian cells involves close coordination of cellular processes. During recent years it has become evident that the sorting, trafficking and distribution of transport vesicles and mRNA granules/complexes are closely coordinated to ensure effective simultaneous handling of all components required for a specific function, thereby minimizing the use of cellular energy. Identification of proteins acting at the crossroads of such coordinated transport events will ultimately provide mechanistic details of the processes. Annexins are multifunctional proteins involved in a variety of cellular processes associated with Ca^2+^-regulation and lipid binding, linked to the operation of both the endocytic and exocytic pathways. Furthermore, certain Annexins have been implicated in the regulation of mRNA transport and translation. Since Annexin A2 binds specific mRNAs via its core structure and is also present in mRNP complexes, we speculated whether direct association with RNA could be a common property of the mammalian Annexin family sharing a highly similar core structure.

**Methods and results:** Therefore, we performed spot blot and UV-crosslinking experiments to assess the mRNA binding abilities of the different Annexins, using annexin A2 and c-myc 3′UTRs as well as c-myc 5′UTR as baits. We supplemented the data with immunoblot detection of selected Annexins in mRNP complexes derived from the neuroendocrine rat PC12 cells. Furthermore, biolayer interferometry was used to determine the K_D_ of selected Annexin-RNA interactions, which indicated distinct affinities. Amongst these Annexins, Annexin A13 and the core structures of Annexin A7, Annexin A11 bind c-myc 3′UTR with K_D_s in the nanomolar range. Of the selected Annexins, only Annexin A2 binds the c-myc 5′UTR indicating some selectivity.

**Discussion:** The oldest members of the mammalian Annexin family share the ability to associate with RNA, suggesting that RNA-binding is an ancient trait of this protein family. Thus, the combined RNA- and lipid-binding properties of the Annexins make them attractive candidates to participate in coordinated long-distance transport of membrane vesicles and mRNAs regulated by Ca^2+^. The present screening results can thus pave the way for studies of the multifunctional Annexins in a novel cellular context.

## 1 Introduction

Annexins (Anxs) are multi-functional and -compartmental proteins participating in a variety of cellular functions related to cell proliferation, membrane-cytoskeleton interactions, endo- and exocytosis, the formation of exosomes, as well as mRNA transport and translation (Vedeler and Hollas, 2000; Filipenko et al., 2004; Gerke et al., 2005; Vedeler et al., 2012; Bharadwaj et al., 2013). Membrane repair is another cellular function that several Anxs such as AnxA1, AnxA2, AnxA4, AnxA5, AnxA6 and AnxA7 are involved in (Draeger et al., 2011; Boye and Nylandsted, 2016a; Boye and Nylandsted, 2016b; Häger and Nylandsted, 2019; Koerdt et al., 2019; Sønder et al., 2019; Croissant et al., 2022; Mularski et al., 2022). Thus, the Anxs may function as hubs and sensors for the coordination of several cellular processes (Monastyrskaya, 2018). Twelve Anxs and splice variants thereof have been identified in mammals, displaying considerable interspecies conservation (Rescher and Gerke, 2004; Gerke et al., 2005). In addition, it is likely that some of the functional roles of the Anxs are redundant and may partially be shared by other members of the family (Rescher and Gerke, 2004; Aareskjold et al., 2019).

The key functional criteria for being assigned as a member of Anx family is that a protein has the ability to bind negatively charged phospholipid-containing membranes in a Ca^2+^-dependent manner (Gerke and Moss, 2002; Moss and Morgan, 2004; Gerke et al., 2005). An exception to this criterion is AnxA10, which is unable to bind to liposomes containing negatively charged phospholipids at physiological Ca^2+^ concentrations and was suggested to be involved in RNA-related processes (Quiskamp et al., 2014). Another Anx criteria is that the protein in question must contain the well-known structurally conserved C-terminal core structure with repetitive structural domains (Gerke et al., 2005). Thus, the typical C-terminal core structure of an Anx consists of four (AnxA6 has eight) domains of ∼70 amino acid residues (domains I-IV), which each harbor five right-handed α-helices (helices A-E). Helices A and B as well as D and E are anti-parallel pairs that are linked by loops, whereas helix C functions as a link between the two pairs of helices. Each domain contains a conserved Anx signature region called the endonexin fold (Geisow et al., 1986). The N-terminal region of the proteins varies in length and composition and is the main element important in the functional differences among the Anx family members (Gerke and Moss, 2002; Bharadwaj et al., 2013). Furthermore, post-translational modifications of the N-terminus add another layer of functional diversity between the different Anxs, as well as for the individual Anx protein (Lauvrak et al., 2005; Nazmi et al., 2012; Grindheim et al., 2014; Caron et al., 2015; Grindheim et al., 2017). Although the Anx proteins possess similar core structures, they show only 45%–55% sequence identity among each other and 25%–30% identity between the domains of the protein core (Barton et al., 1991; Moss and Morgan, 2004). Thus, different members of the Anx family may bind specific ligands not only via their N-terminus, but also via their core structure. Although actin interacts with several members of the Anx family such as AnxA1, AnxA2, AnxA6 and AnxA8 via their core structure, this does not appear to be a universal Anx trait (Hayes et al., 2004; Rescher and Gerke, 2004; Goebeler et al., 2006; Hayes et al., 2006; Patel et al., 2011; Demonbreun et al., 2016). Interestingly, as mentioned, one member of the Anx family, AnxA10, does not exhibit the Ca^2+^- and lipid-dependent binding to membranes, which is characteristic for the other mammalian Anxs, but co-localizes with the two mRNA-binding proteins, splicing factor proline- and glutamine-rich (SFPQ) and paraspeckle component 1 (PSPC1) to paraspecles in the nucleus (Quiskamp et al., 2014).

We earlier discovered that AnxA2 acts as an mRNA-binding protein associated with a specific subpopulation of mRNP complexes linked to the cytoskeleton (Vedeler and Hollas, 2000; Mickleburgh et al., 2005; Aukrust et al., 2006; Hollas et al., 2006). In addition, we mapped the mRNA-binding site of AnxA2 to helices C-D of its domain IV in the core structure (Aukrust et al., 2007) and identified AnxA2-binding regions in the 3′untranslated regions (UTRs) of anxA2 (Hollas et al., 2006) and c-myc (Mickleburgh et al., 2005) mRNAs. An apparent K_D_ value of ∼8 nM at steady state was estimated for the AnxA2-5′GGGGAUUG interaction (Solbak et al., 2017). AnxA2 also binds the 3′UTR of N-methyl-d-aspartate receptor 1 (Anji and Kumari, 2011) and the collagen prolyl-4-hydroxylase-α(I) subunit (Fahling et al., 2006) mRNAs. It binds to the internal ribosome entry sites (IRES) of p53 and increases the synthesis of the N-terminally truncated isoform of p53 in a Ca^2+^-dependent manner (Sharathchandra et al., 2012). Furthermore, it binds the IRES of c-myc mRNA, also in a Ca^2+^-dependent manner, inhibiting the translation of the c-myc mRNA (Strand et al., 2021). AnxA2 interacts with NS5B from hepatitis C virus and both proteins bind RNA, although with different preferences (Solbak et al., 2017). Interestingly, AnxA2 binds coronavirus RNA and reduces its frameshifting efficiency and thus presents anti-viral activities against these viruses (Kwak et al., 2011). AnxA1 and AnxA11 are other examples of RNA-binding Anxs (Hirata and Hirata, 1999; Liao et al., 2019). Otherwise, very little is known about the mRNA-binding properties of the Anx family.

Eukaryotic mRNAs are organized in mRNP complexes (also called mRNA granules) together with proteins and regulatory RNAs (Nabariya et al., 2022; Vorländer et al., 2022). The protein components of the mRNA granules control mRNA transport, stability, anchorage and translation (Protter and Parker, 2016; Khong and Parker, 2020). These proteins may bind directly or indirectly to mRNA sequences forming mRNP complexes (Khong and Parker, 2020), where their interactions with both mRNAs and other proteins are regulated by post-translational modifications. Some RNA-binding proteins are capable of non-sequence specific binding to RNA while others recognize specific target sequences (Glisovic et al., 2008). AnxA2 has been found to bind directly to distinct RNA sequences within the 3′UTRs of anxA2 and c-myc mRNAs containing higher order structures with a five nucleotide consensus sequence 5′AA (C/G) (A/U)G (Mickleburgh et al., 2005; Hollas et al., 2006). These are mRNAs translated on cytoskeleton-bound polysomes (Vedeler and Hollas, 2000; Vedeler et al., 2012). We have previously described the high-molecular-mass modified forms of AnxA2 associated with mRNP complexes (Lauvrak et al., 2005; Aukrust et al., 2017). Ser25 phosphorylation and ubiquitin/SUMO1 conjugation of AnxA2 target the protein for binding to non-polysomal mRNAs (Aukrust et al., 2017). This may be a general post-translational modification which targets Anxs to establish RNA interactions. We have previously noted that Ser25 phosphorylated AnxA2 partially colocalizes with the P-body marker GW182 (Aukrust et al., 2017), while AnxA1, AnxA6, AnxA7 and AnxA11 have been identified in stress granules. Stress granules and P-bodies are spatially, compositionally and functionally linked complexes of stalled translationally inactive mRNAs (Kedersha et al., 2005).

The mRNAs are transported in large complexes often consisting of a set of mRNAs participating in the same cellular process (Vargas et al., 2022). Some proteins are relatively ubiquitous while other proteins may define sub-populations of mRNA granules. Interestingly, the intracellular distribution mechanisms of the membrane-less mRNP complexes are linked to membrane trafficking as it is becoming increasing clear that mRNA transport and translation involve different types of organelle and vesicle dynamics (Müntjes et al., 2021; Vargas et al., 2022). Accordingly, membrane traffic and mRNA transport appear as coordinated processes responding to shared extracellular signals derived from common pathways. Thus, there is a quest to reveal proteins that may link these two processes. AnxA11 that functions both as an mRNA- and lipid-binding protein has been introduced as a candidate to co-ordinate these two processes (Liao et al., 2019).

Recent findings suggest a novel form of intercellular communication based on the direct transfer of genetic information (mRNA, non-coding RNA) in small vesicles known as exosomes, which represents a mode of epigenetic regulation of gene expression. Exosomes are a special type of secretory membrane vesicles (Simons and Raposo, 2009), released to the extracellular space from multivesicular bodies (MVBs) that fuse with the plasma membrane (PM) (Lachenal et al., 2011). Other types of extracellular vesicles also exist that appear to function in a similar manner (O’Brien et al., 2020). All Anxs, except AnxA9 and AnxA10, have so far been found in exosomes (http://www.exocarta.org/) (Gurung et al., 2021). AnxA2 is the best characterized member of the Anx family both in terms of its association with mRNA (Vedeler and Hollas, 2000; Vedeler et al., 2012; Strand et al., 2021; Grindheim et al., 2023) and exosomes (Valapala and Vishwanatha, 2011; Grindheim et al., 2016; Grindheim and Vedeler, 2016; Desai et al., 2022). AnxA2 appears to play a role in loading RNA cargo into exosomes (Monastyrskaya, 2018). Regulatory RNAs that bind to the regulatory regions of anxA2 mRNA as well as anxA2 mRNA are incorporated into exosomes together with AnxA2 (Monastyrskaya, 2018; Desai et al., 2022) strongly suggesting that exosomes carry information to regulate the expression of AnxA2 in the recipient cell(s) since anxA2 mRNA binds to its cognate protein (Hollas et al., 2006). At present, the functional role of the other Anxs associating with exosomes has not been elucidated.

In addition to proteins and specific mRNAs, exosomes may transfer soluble factors and miRNAs (Valadi et al., 2007) and even virus particles (Wiley and Gummuluru, 2006) between cells. Interestingly, findings indicate that exosomes are internalized by the recipient rat neuroendocrine PC12 cells by endocytosis and transported to the perinuclear region along cytoskeletal tracks. Subsequently, exosomal lipids are recycled to the PM while most of the protein components are targeted to lysosomes for degradation (Tian et al., 2010). These findings strongly suggest that exosomal cargo, in particular specific mRNAs, are released to the cytoplasm in the perinuclear region and then delivered directly to ribosomes for translation or re-directed to other cellular sites for local translation. Interestingly, AnxA2 is involved in the transport of specific mRNAs to the perinuclear region for local translation (Veyrune et al., 1996; Mickleburgh et al., 2005). AnxA2 binds transiently to the eukaryotic initiation factor eIF4F thereby inhibiting translation (Grindheim et al., 2023). Thus, it may be involved in the transport of mRNAs in their silent form in a process which is regulated by its post-translational modifications. Since very little is known about the function of the other members of the Anx family in RNA-related processes, it remains an interesting subject of future studies.

Since our initial experiments analyzing the interaction between AnxA2 and mRNA showed that the RNA-binding site is located in domain IV of the conserved structural core, we speculated that the RNA-binding property could be a common feature of several Anxs, if not shared by all of them. To investigate this possibility, we cloned all rat Anxs and investigated their ability to bind mRNA directly, both to oligo (dT) affinity-purified total mRNA from PC12 cells and *in vitro* transcribed regions of specific mRNAs. The obtained results are supported by the determination of the K_D_s of selected Anxs.

## 2 Materials and methods

### 2.1 Cloning of the rat anx cDNAs

To obtain the full-length rat Anxs, RT-PCR was performed using total RNA isolated from PC12 cells by the Trizol method (Chomczynski and Sacchi, 1987). The rat anxA8 and anxA13 cDNAs were generated from total RNA derived from rat lung (a generous gift from Dr. Ingeborg Winge) and rat colon (LS-J1759; LSBio, Seattle, United States), respectively. Rat anxA9 cDNA in the pET-16b vector was purchased from GenScript Biotech (Piscataway, United States) and subcloned into the pETM10 vector (a kind gift from Dr. Gunter Stier, Heidelberg). The cDNAs of the Anxs (except anxA9 cDNA) were obtained using the iScript kit (Bio-Rad; Hercules, United States) according to the manufacturer’s instructions and the forward and reverse primers as listed in Table 1. All primers were purchased from Sigma (Merck/MilliporeSigma; St. Louis, United States). The anx cDNAs were digested with *Nco*I and *Acc*65I (or *Bsa*I to produce *Nco*I compatible sites; the *Not*I restriction enzyme was used instead of *Acc*65I for anxA6 and anxA13 cDNAs due to the presence of internal *Acc*65I cleavage sites), purified after separation in a 1% agarose gel and ligated into the same restriction sites of the pETM10 vector. All restriction enzymes used were FastDigest Restriction Enzymes (Thermo Scientific; Waltham, United States). N-terminally truncated versions of AnxA7 (Δ142AnxA7) and AnxA11 (Δ188AnxA11) (Lillebostad et al., 2020) were generated by PCR amplification using the pETM10 plasmid harboring rat full-length anxA7 and anxA11 cDNA, respectively, as templates. This resulted in AnxA7 and AnxA11 lacking 142 amino and 188 amino acids, respectively, at the N-terminally end. The same reverse primer was also used for the full-length constructs. All constructs were verified by DNA sequencing, performed at the Section for Medical Genetics and Molecular Medicine at Haukeland University Hospital, Bergen, Norway.

**TABLE 1:** Sequence of primers and identity of sequenced clones of rat anx cDNAs. The colored boxes represent recognition sites for restriction enzymes. Yellow, blue, green and red colors highlight the *Nco*I, *Acc*65I, *Not*I and *Bsa*I sites, respectively. Letters in bold represent NcoI compatible sites.

**Rat anxA1 isoform X1 cDNA (346 aa) (XP_008758517.1 GI: 672039949):**
Forward 5′-ATCCGG CCATGG GTATGGCAATGGTATCAGAATTC
Reverse 5′-ATCCGG GGTACC TTAGTTTCCTCCACACAGAG
**Rat anxA2 cDNA isoform CRA_a (339 aa) (gi|149028869|gb|EDL84210.1|):**
Forward 5′-ATCCGG CCATGG GTATGTCTACTGTCCACGAAATC
Reverse 5′-ATCCG GGTACC TCAGTCGTCACCACCACACAG
**Rat anxA3 isoform CRA_b cDNA (324 aa) (>gi|149046863|gb|EDL99637.1|):**
Forward 5′-ATCCGG CCATGG GTATGGCGGCGTCTTTGTGGGTTG
Reverse 5′-ATCCGG GGTACC TCAATCATCTCCTCCACAGATC
**Rat anxA4 isoform CRA_a (X1) cDNA (319 aa) (>gi|149036642|gb|EDL91260.1|):**
Forward 5′-ATCCGG CCATGG GTATGGAAACCAAAGGAGGAACTG
Reverse 5′-ATCCGG GGTACC TTAATCATCTCCTCCACAGAG
**Rat anxA5 isoform CRA_a cDNA (319 aa) (>gi|149048734|gb|EDM01275.1|):**
Forward 5′-ATCCGG CCATGG GTATGGCTCTCAGAGGCACCGTG
Reverse 5′-ATCCGG GGTACC CTCAGTCATCCTCGCCTCCACAG
**Rat anxA6 cDNA (673 aa) (>gi|130502086|ref|NP_077070.2|):**
Forward 5′-ATCCGG CCATGG GTATGGCTAAAATAGCACAGGGTGC
Reverse 5′-ATCCGG GCGGCCGC TTAGTCTTCTCCACCACAGAGC
**Rat anxA7 isoform X1 cDNA (463 aa) (>gi|672077397|ref|XP_008768707.1):**
Forward 5′-ATCCGG GGTCTC TCATGGGTATGTCATATCCAGGCTACC
Reverse 5′-ATCCG GGGTACC TCACTGGCCAACGATTGCTAG
**Rat Δ142anxA7 isoform X1 cDNA (>gi|672077397|ref|XP_008768707.1):**
Forward 5′-ATCCGG GGTCTC TCATGGGTCCTGCTGCGATGACT
Reverse 5′-ATCCGG GGTACC TCACTGGCCAACGATTGCTAG
**Rat anxA8 cDNA (327 aa) (>gi|72255533|ref|NP_001026824.1|):**
Forward 5′-ATCCGG GGTCTC TCATGGGTATGGCCTGGTGGAAAGCCTGG
Reverse 5′-ATCCGG GGTACC TCAAAGGTCAGTACCAACAAGG
**Rat anxA9 isoform X1 cDNA (369 aa) (>gi|564337832|ref|XP_006232999.1|):**
Forward 5′-ATCCGG GGTCTC TCATGGGTATGTCTGTGAGCTGTGGAC
Reverse 5′-ATCCGG GGTACC TCAGATGTCTTCTGCTCTACAC
**Rat anxA10 cDNA (324 aa) (>gi|1,57819523|ref|NP_001102580.1|):**
Forward 5′-ATCCGG CCATGG GTATGTTCTGTGGGGAATATGTCC
Reverse 5′-ATCCGG GGTACC TTAATAGTCTTCCACATCACCG
**Rat anxA11 cDNA (503 aa) (>gi|58865414|ref|NP_001011918.1|):**
Forward 5′-ATCCGG CCATGG GTATGAGCTATCCAGGCTATCCAC
Reverse 5′-ATCCGG GGTACC TCAGTCGTTGCCACCACAGATC
**Rat Δ188anxA11 cDNA (>gi|58865414|ref|NP_001011918.1|):**
Forward 5′-ATCCGG CCATGG GTAGAGGCACCATCA
Reverse 5′-ATCCGG GGTACC TCAGTCGTTGCCACCACAGATC
**Rat anxA13 cDNA (319 aa) (>gi|201027432|ref|NP_001128382.1|):**
Forward 5′-ATCCGG GGTCTC TCATGGGTATGGGGAATCATCATGCCAAAG
Reverse 5′-ATCCGG GCGGCCGC TCAGTGCAAGAGTGCTACCAGC

### 2.2 Expression and purification of recombinant Anxs

*E. coli* BL21 (DE3) bacteria were transformed with pETM10 plasmids containing the respective anx cDNAs. The bacteria were grown in 300 mL LB medium with 50 μg/mL kanamycin until they reached an OD600 of 0.6. Subsequently, 1 mM isopropyl β-D-1-thiogalactopyranoside (IPTG) was added to induce protein expression. The N-terminally truncated versions of AnxA7 and AnxA11 were both induced at 25°C for 4 h, while wt Anxs were expressed ON at 15°C. Following incubation, the bacteria were collected by centrifugation at 4,000 *g* for 15 min. The pellets were frozen at −80°C and thawed in Breakage buffer (50 mM Na_2_HPO_4_; pH 8.0, 500 mM NaCl, 10 mM imidazole, 5% (w/v) glycerol, 1 mM DTT) before the addition of 0.5 μg/mL DNase I, 0.25 μg/mL RNase A and EDTA-free protease inhibitor cocktail (cOmplete, Roche; Basel, Switzerland). Subsequently, the bacteria were sonicated on ice 8 × 30 s and the lysates were centrifuged at 16,000 *g* for 30 min at 4°C. All purification steps were performed at 4°C. The supernatants from the cleared bacterial lysates were loaded onto Ni^2+^-NTA or Co^2+^-NTA (in case of AnxA7, AnxA9 and AnxA11) agarose columns and incubated for 30 min on a turning wheel. Purification on Co2+^2+^ instead of Ni^2+^ resin resulted in less impurities in the AnxA7, AnxA9 and AnxA11 protein samples. Purification of the Anxs was performed essentially as described (Lillebostad et al., 2020). All Anxs were subjected to size exclusion chromatography using a Superdex 75 or 200 Increase 10/300 GL column (GE Healthcare, Chicago, IL, United States), except for wt AnxA7, wt AnxA9 and wt AnxA11 due to their instability and low yield. Protein concentration of the recombinant proteins was determined by absorbance at 280 nm (using molecular masses and sequence-based extinction coefficients). The proteins were concentrated (except for wt AnxA7, wt AnxA9 and wt AnxA11) and quick frozen in liquid nitrogen and stored in aliquots at −80°C until usage. The correct protein sizes were confirmed by SDS-PAGE and Coomassie Brilliant blue staining. Note that wt AnxA9, but also to some degree wt AnxA7 and wt AnxA11 were notoriously bad-behaving proteins prone to aggregation and degradation.

### 2.3 Culture of PC12 cells

The rat adrenal pheochromocytoma PC12 cells, representing a readily adherent sub-clone derived from the original PC12 cell line (Greene and Tischler, 1976) (a generous gift from Prof. Jaakko Saraste), were grown in RPMI 1640 medium supplemented with 10% (v/v) heat-inactivated horse-serum, 5% (v/v) fetal bovine serum, 2 mM L-glutamine, 100 units penicillin/mL and 100 µg streptomycin/mL. As described previously (Grindheim et al., 2014), the cells were routinely cultured at 37°C in a humidified atmosphere of 21% O_2_ supplemented with 5% CO_2_.

### 2.4 Subcellular cell fractionation and isolation of polysomes as well as mRNP complexes

The cytosolic, cytoskeletal and membrane fractions of PC12 cells were isolated essentially as described previously (Vedeler and Hollas, 2000; Aukrust et al., 2017). Polysomes present in the cytoskeletal and membrane-bound fractions were isolated by centrifugation through a 35% (1 M) sucrose cushion for 2 h at 100,000 g and finally resuspended in 130 mM KCl buffer (Aukrust et al., 2017). Polysomes were stabilized by the addition of cycloheximide (CHX) to the culture medium at a final concentration of 100 μg/mL for 15 min before harvesting of cells (Bensaude, 2011). Protein determinations were done by IR measurements in triplicates.

The cytoskeletal fraction (∼800 μg protein) of PC12 cells was diluted 1:4 in RNA-binding buffer (10 mM triethanolamine (pH 7.4), 50 mM KCl, 1 mM DTT, 2 mM MgSO_4_, 100 µM CaCl_2_) containing 1 mg/mL yeast tRNA and 0.4 U/µL RNasin (Promega; Madison, United States) or Ribolock RNase inhibitor (Fermentas, Thermo Fisher Scientific; Waltham, United States). Subsequently, the fraction was incubated for 60 min with oligo (dT) magnetic beads (Dynal, Thermo Fisher Scientific; Waltham, United States) at 4°C on a turning wheel. The beads were then washed three times with tRNA-free RNA-binding buffer. Polyadenylated mRNAs with bound proteins were eluted from the beads by incubation for 10 min at 65°C in 60 µL elution buffer (pre-heated at 65°C) containing 0.1% SDS and 1 mM DTT. The elution step was repeated once, and the mRNAs in the two combined fractions were degraded by incubation with 0.3 μg/μL RNase A for 10 min at 30°C to facilitate the separation of proteins by SDS-PAGE for immunoblot analyses.

### 2.5 Metabolic labeling and subsequent isolation of total RNA and total mRNA from PC12 cells

Confluent PC12 cells (5 x T75 flasks) were incubated for 60 min in a phosphate-free medium (Thermo Fisher Scientific; Waltham, United States) supplemented with dialyzed 15% fetal calf serum and 5 mCi [^32^P] inorganic phosphate (specific Activity: 8,500–9,120 Ci; 10 mCi/mL; PerkinElmer) per T75 flask. The medium was poured off and the cells were resuspended in 9 mL Trizol reagent. In the next step, the suspension was transferred to a Corex tube, incubated for 5 min at RT and 2 mL chloroform was added per mL Trizol. The sample was shaken vigorously by hand and incubated for 3 min at RT before centrifugation at 12,000 *g* for 15 min at 4°C. The aqueous phase was transferred into a new Corex tube, and the radiolabeled RNA was precipitated by using 0.5 mL isopropanol per mL Trizol and incubated for 10 min at RT. The precipitated RNA was collected by centrifugation at 12,000 *g* for 10 min at 4°C. The supernatant on top of the RNA pellet was discarded. Subsequently, the RNA pellet was washed in cold 70% (v/v) EtOH and centrifuged at 7,500 *g* for 5 min at 4°C. After drying, the RNA pellet was dissolved in 30 μL ddH_2_0 and incubated for 10 min at 70°C. The sample with total RNA was divided in smaller aliquots and stored at −80°C. Total mRNA was affinity-purified from total RNA using Dynabeads (dT)25 (Thermo Fisher; Waltham, United States) according to the procedure given by the manufacturer.

### 2.6 Cloning and *in vitro* transcription of renilla luciferase, anxA2 and c-myc transcripts

*In vitro* transcription was performed on PCR amplified cDNA fragments to produce transcripts (both radiolabeled and non-labeled), taking advantage of the T7 promoter site introduced by the forward primer. The 1,356 bp form of full-length rat anxA2 cDNA (including sequences coding for the UTRs) was obtained by RT-PCR using total RNA isolated from PC12 cells as previously described and cloned into the pGEM3Zf (+) vector (Aareskjold et al., 2019). The cDNA PCR fragment with a T7-promoter in front of full-length anxA2 mRNA was obtained using the pGem3Zf (+) vector with the anxA2 cDNA insert as template, the forward primer (5′GAA​ATT​AAT​ACG​ACT​CAC​TAT​AGG-GAG​GCT​CTC​TGC​AAT​AGG​TGC) of the 5′UTR of rat anxA2 cDNA containing the T7 promoter sequence and the rat anxA2 3′UTR reverse primer (5′AAA​GTA​AAA​TGG​TTT​ATT​C), while the rat anxA2 3′UTR transcript was obtained by linearizing the pGEM3Zf (+) vector containing the corresponding cDNA insert with *Bam*HI. The coding sequence (CDS) of humanised Renilla Luciferase cDNA (hRLuc; 936 bp) was produced by PCR amplification of the sequence in the phRL-CMV vector (Promega, Madison, United States) using the forward primer 5′TAA​TAC​GAC​TCA​CTA​TAG​G-ATG​GCT​TCC​AAG​GTG​TAC​GA and the reverse primer, 5′TTA​CTG​CTC​GTT​CTT​CAG​CA. c-myc 3′UTR PCR products was produced using the forward primer, 5′TAA​TAC​GAC​TCA​CTA​TAG​G-ACT​GAC​CTA​ACT​CGA​GGA​GG and the reverse primer 5′GTA​TTT​TTT​CCA​ATT​ATT​TT and the mouse c-myc cDNA in the pGEM3Zf (+) vector generated by RT-PCR from mouse cells (a generous gift from Prof. Silke Appel, University of Bergen). PCR products were obtained using the ACCUtaq DNA polymerase (Sigma-Aldrich; Merck; Darmstadt, Germany). The generation of the 5′UTR of the c-myc mRNA has been described previously (Strand et al., 2021). The constructs were verified by DNA sequencing, performed at the Section for Medical Genetics and Molecular Medicine at Haukeland University Hospital, Bergen, Norway. The PCR products were subjected to electrophoresis and purified from a 1% (w/v) agarose gel using Wizard SV Gel and PCR Clean-Up System (Promega; Madison, United States). The purified cDNA templates were used *in vitro* transcription assays using the HiScribe T7 High Yield RNA Synthesis Kit (New England BioLabs; Ipswich, United States) according to the manufacturer for non-radiolabeled transcripts. The mRNA was extracted using the BioUltra phenol:chloroform:isoamyl alcohol (125:24:1; pH 4–5) (Sigma-Aldrich, Merck; Darmstadt, Germany) and BioUltra chloroform:isoamyl alcohol (24:1) (Sigma-Aldrich, Merck; Darmstadt, Germany) method. Subsequently, the mRNA was precipitated with 2.5 volumes of 96% ethanol (Vinmonopolet, Oslo, Norway) and 3 M sodium acetate (pH 5.2; Thermo Fisher Scientific; Waltham, United States). Finally, after washing in 70% ethanol, the mRNA was resuspended in ddH_2_O and stored in small aliquots at −80°C. *In vitro* transcription of radiolabeled RNA transcripts was performed on the PCR amplified cDNA fragments as described above using the RiboMax Large Scale RNA Production System T7 (Promega; Madison, United States). Radioactive [α-^32^P]-rUTP (3,000 Ci/mmol; 10 mCi/mL EasyTide; PerkinElmer; Waltham, United States) was incorporated during transcription to label the transcripts. The RNA transcripts were subjected to agarose gel electrophoresis to confirm the transcription of correctly sized RNAs and to ensure that the RNAs were not degraded. For details, see (Hollas et al., 2006). Typically, a specific activity of ∼1.5–2 x 10^8^ cpm/μg transcript was achieved.

### 2.7 Determination of RNA-protein interactions using spot blot analysis

The RNA-binding abilities of the Anx proteins were studied by exposing nitrocellulose membranes spotted with the twelve rat Anx proteins in their native form to [^32^P] metabolically labeled total RNA or total mRNA derived from rat PC12 cells or *in vitro* transcribed [^32^P] rUTP-labeled transcripts of anxA2 mRNA (full-length), c-myc 3′UTR or the CDS of hRLuc mRNA. Protein samples (3 µL) were spotted onto nitrocellulose membranes (0.2 µm pore size) in three different concentrations (5, 10 and 20 µM) and air-dried. The samples of AnxA2 that were denatured, were heat-inactivated at 95°C for 5 min. The membranes were incubated overnight in 1xDenhard’s solution [0.022% each of Ficoll, polyvinylpyrrolidone and bovine serum albumin (BSA)] in RNA-binding buffer (10 mM triethanolamine; pH 7.4, 50 mM KCl, 1 mM DTT, 2 mM MgSO_4_, 100 µM CaCl_2_) containing tRNA (1 mg/mL) to block unspecific binding of RNA. The RNA transcripts (200,000 cpm) were preheated for 3 min at 72°C, and then gradually cooled for 15 min to RT to allow folding of secondary structure and subsequently incubated together with (1 u/mL) RiboLock RNase Inhibitor (Thermo Fisher Scientific; Waltham, United States) with the proteins on the spot blot for 30 min at RT on a tilting disk. The RNA-binding buffer containing radiolabeled RNA was removed and the membranes were quickly rinsed three times with RNA-binding buffer without tRNA. The membranes were further washed 4 × 15 min in the same buffer. The membranes were air-dried for 30 min and exposed to phosphor-imager screens (Fuji, Tokyo, Japan). Bound RNA was detected by scanning in a Phosphor-Imager (Bas-5000; Fuji; Tokyo, Japan).

### 2.8 Determination of RNA-protein interactions using UV-crosslinking experiments

The interaction of the Anxs with the RNAs (*in vitro* transcribed [^32^P] rUTP-labeled transcripts of anxA2 or c-myc 3′UTRs) was assayed by UV-crosslinking experiments using the RNA-binding buffer described in Section 2.7, and performed essentially as described earlier (Mickleburgh et al., 2005; Hollas et al., 2006). The transcripts were preheated to 72°C for 3 min and gradually cooled to RT. The UV-crosslinked RNA-protein complexes were separated by SDS-PAGE after RNAse treatment. The gels were dried and exposed to screens. The RNA-binding abilities of the Anx proteins were studied by scanning the screens in a Phosphor-Imager (Bas-5000; Fuji; Tokyo, Japan). The binding assays of the competition experiments consisted of both radiolabeled and unlabeled competitor RNA.

### 2.9 Determination of Anx-RNA interactions using biolayer interferometry (BLI) experiments

The anxA2 and c-myc 3′UTRs were biotinylated at the 3′-end using periodate-chemistry. In brief, the 2′,3′ diols of the *in vitro* transcribed RNAs were oxidized to aldehydes as described (Rinaldi et al., 2015). 5 μM RNA was incubated with 5 mM NaIO_4_ and 0.1 M NaOAc, pH 5.2 for 60 min. The RNA was precipitated as described in Section 2.6. The resulting RNA pellet was dissolved in 10 mM aqueous solution of EZ-Link^®^ Hydrazide-PEG-Biotin (Thermo Fisher Scientific, Waltham, United States) and incubated at 37°C for 2 h. 0.2 M NaBH_4_ and 1 M Tris-HCl, pH 8.2 were added and the reaction was incubated for 30 min on ice in the dark. The RNA was precipitated as described above and resuspended in ddH_2_O. The RNA samples were immobilized onto High Precision Streptavidin (SAX) biosensors (Sartorius AG, Göttingen, Germany) by dipping into 40 nM RNA solution for 2 min at a stir rate of 1,000 rpm. The reference sensors were prepared by dipping SAX biosensors into 10 µM of biocytin solution. The proteins of interest were prepared in RNA-binding buffer at a dilution series of 0, 0.1, 0.2, 0.4, 0.8, 1.6 and 3.2 µM. Binding was monitored on the OctetRED96 instrument (FortéBio, Menlo Park, United States) by allowing the association for 180 s and the dissociation for 200 s. The data were analyzed for a 1:1 binding model using fortéBIO Octet data analysis software (version 9). Briefly, the response of each RNA immobilized sensor for protein analytes was subtracted with the response for the same sample from the biocytin immobilized reference sensors. This was followed by baseline correction and subsequently applying Savitzky-Golay filtering according to the application note “Biomolecular Binding Kinetics Assays on the Octet^®^ BLI Platform by Sartorius. The kinetic local fitting was applied to an individual group of unique sensor locations using a 1:1 model. The fitness of the data to the model was judged by the reduced χ2 and R^2^ values and K_D_ values were reported for R^2^ values >0.9.

### 2.10 SDS-PAGE and western blot analysis

SDS-PAGE was performed using 10% or 4%–15% gradient gels (Mini-Protean TGX; Bio-Rad) and the denaturation buffer used was from BioRad (Cat. 1610747) added 100 µL β-mercapto ethanol per 900 µL buffer. The composition of the denaturation buffer is 277.8 mM Tris-HCl (pH 6.8), 44.4% (v/v) glycerol, 4.4% LDS, 0.02% bromophenol blue. The proteins were transferred onto nitrocellulose membranes (0.2 µm pore size) by blotting performed using the Trans-Blot Turbo Transfer System (Bio-Rad; Hercules, United States) essentially according to the manufacturer (25V/1.3 mA, 10 min transfer). The membranes were probed against AnxA1 (HPA011272, Sigma-Aldrich, Saint-Louis, United States; 1:1,000), AnxA2 (610069; BD Biosciences, Franklin Lakes, United States; 1:1,000), AnxA4 (PA5-82296, Invitrogen, Thermo Fisher Scientific, Waltham, United States; 1:1,000), AnxA5 (MA5-35789, Invitrogen, Thermo Fisher Scientific, Waltham, United States; 1:1,000), AnxA6 (NBP1-90149, Novus Biologicals LTD., Bristol, UK; 1:1,000), AnxA7 (sc-17815, Santa Cruz Biotechnology, Dallas, United States; 1:1,000), AnxA10 (ab213656, Abcam, Cambridge, UK; 1:1,000), AnxA11 (sc-9322, Santa Cruz Biotechnology, Dallas, United States; 1:1,000), AnxA13 (PA5-109395, Invitrogen, Thermo Fisher Scientific, Waltham, United States; 1:1,000)), Rab7 (R4779, Sigma/Merck,; 1:1,000), Rab11 (610657, BD Transduction Lab/Thermo Fisher Scientific, Waltham, United States; 1:1,000), EEA1 (610457, BD Transduction Lab/Thermo Fisher Scientific, Waltham, United States; 1:1,000), S6 (710405; Thermo Fisher Scientific; Waltham, United States; 1:1,000), and PABP1 (4992S; Cell Signaling Technology, Danvers, United States; 1:1,000) primary antibodies. Rabbit polyclonal antibodies against SPC25 was a generous gift from Stephen High (University of Manchester, UK). Proteins were detected by incubation at a 1:2,000 dilution with secondary horseradish peroxidase (HRP)-conjugated antibodies (170–6,516 (goat anti-mouse) or 170–6,515 (goat anti-rabbit) from Bio-Rad, Hercules, United States) or from Santa Cruz, Dallas, United States (sc-2020; donkey anti-goat). The reactive protein bands were visualized using the WesternBright ECL HRP substrate (Advansta; San Jose, United States).

## 3 Results

### 3.1 The presence of Anxs in polysomes and non-polysomal mRNP complexes (RNA granules)

Our previous studies revealed that AnxA2 is enriched in the cytoskeletal fraction. Furthermore, we showed that a subpopulation of AnxA2 in this fraction is associated with mRNAs in polysomes and monosomes (Vedeler and Hollas, 2000) and particularly with translationally inactive mRNP complexes (Vedeler et al., 2012; Aukrust et al., 2017). We have employed PC12 cells in most of our earlier AnxA2 studies and are very familiar with their behavior and morphology (Grindheim et al., 2014; Grindheim et al., 2016; Grindheim and Vedeler, 2016; Aukrust et al., 2017; Strand et al., 2021). Therefore, it was of interest to employ these cells, in which the level of AnxA2 can be upregulated up to 14-fold by NGF stimulation (Fox et al., 1991; Jacovina et al., 2001). We have also used this cell line to test several antisense RNAs and ribozymes against AnxA2 and found that upon AnxA2 knock-down, the level of AnxA7 increases several fold (Aareskjold et al., 2019). In addition, most of the recombinant Anxs were cloned via RT-PCR from PC12 cells.

**FIGURE 1:**
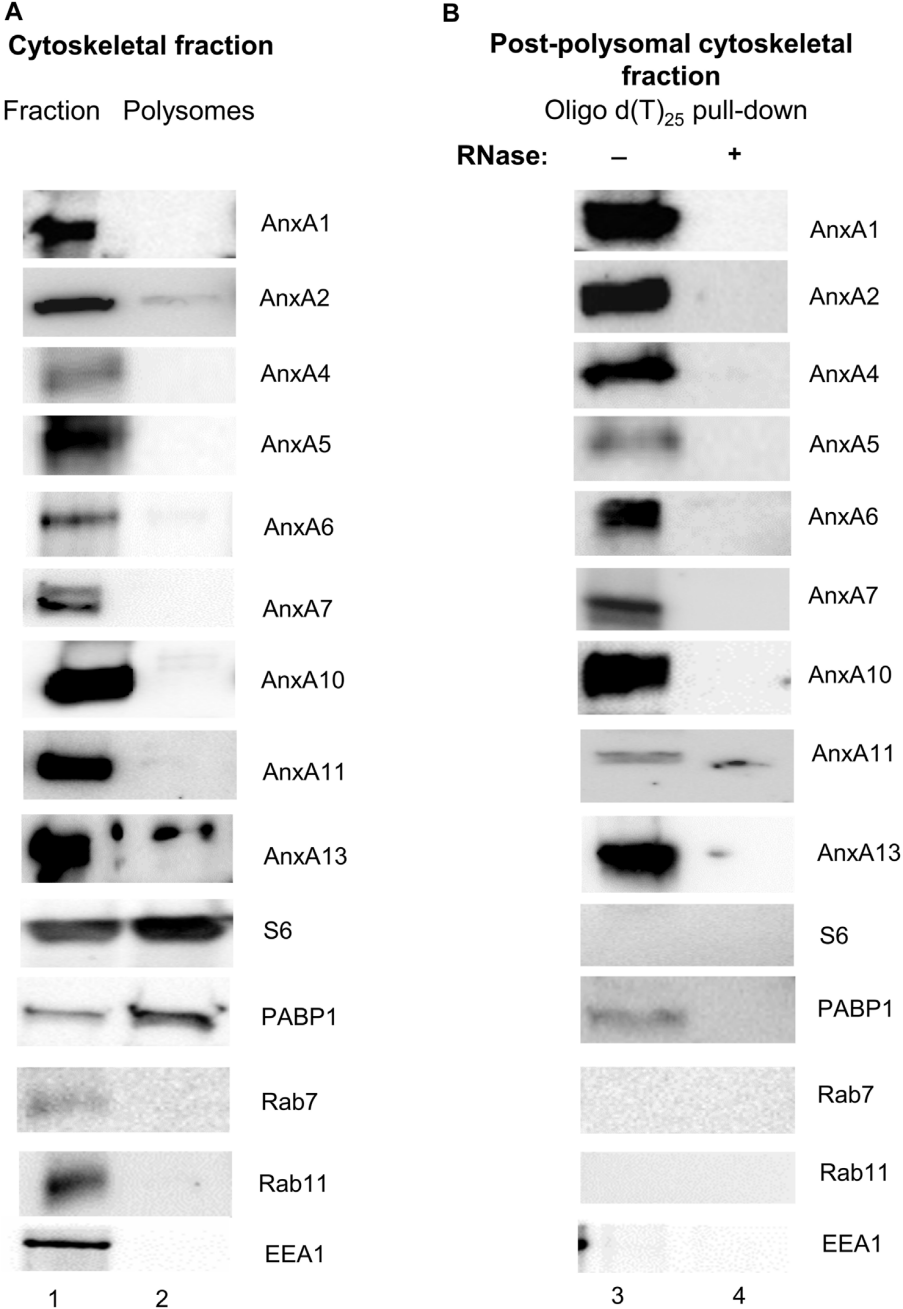
AnxA1, AnxA2, AnxA4, AnxA5, AnxA6, AnxA7, AnxA10, AnxA11 and AnxA13 present in the cytoskeleton fraction (Panel A) are associated with non-polysomal mRNP complexes (Panel B) of PC12 cells. Panel **(A)** 30 µg of the cytoskeletal fraction (lane 1) and cytoskeleton-bound polysomes (lane 2) were separated by 10% SDS-PAGE and transferred to nitrocellulose membranes. Panel **(B)** samples prepared from oligo (dT)-bound mRNP complexes from the cytoskeletal fraction [supernatant after centrifugation for 2 h 100,000 g above a 1 M (35%) sucrose cushion] (lanes 3 and 4), without (lane 3) or with RNase (lane 4) treatment, as indicated above the Western blots, were subjected to similar analysis. The blots were probed with antibodies against the different Anxs and against PABP1 as a marker for poly(A)-containing mRNAs, as indicated. Antibodies against the ribosomal subunit S6 were used to inform of the distribution of ribosomes. In addition, the blots were probed with antibodies against early endosomes (EEA1), late endosomes (Rab7) and recycling endosomes (Rab11). SPC25 and LAMP1 were not detectable in any of the fractions (results not shown). Visualization of the immunoreactive protein bands was performed using the ChemiDocTM XRS+ molecular imager after incubation with horseradish peroxidase (HRP)-conjugated secondary antibodies and enhanced chemiluminescence (ECL)-reagent. The blots shown are representative for results from three experiments.

Thus, we first isolated polysomes from the cytoskeletal fraction (Vedeler et al., 1991; Aukrust et al., 2017) derived from PC12 cells and using specific antibodies against AnxA1, AnxA2, AnxA4, AnxA5, AnxA6, AnxA7, AnxA10, AnxA11 and AnxA13 investigated their presence in the purified polysome fractions and oligo-d(T) purified mRNP complexes from the post-polysomal cytoskeletal fraction. The polysomes were pelleted through a 35% (1 M) sucrose cushion (Doller et al., 2013) to avoid the pelleting of Anx-containing membrane vesicles such as endosomes, which due to their lipid content have a low density (de Araujo et al., 2015). Lysosomal-associated membrane protein 1 (LAMP1) and SPC25, a protein present in the endoplasmic reticulum (Wilson et al., 2005), were not present in the cytoskeletal fraction (results not shown). Rab7 (marker for late endosomes), Rab11 (marker for recycling endosomes) and early endosome autoantigen 1 (EEA1; marker for early endosomes) were found in the cytoskeleton fraction (Figure 1A). This suggests that the 1 M sucrose cushion effectively separates vesicles from polysomes and mRNP complexes. This was expected since this fractionation protocol was developed to isolate free, cytoskeleton-bound and membrane-bound polysomes (Vedeler et al., 1991). Only minor fractions of the Anxs were detectable in the cytoskeletal polysomes, which is most evident in the case of AnxA2 and AnxA13 (Figure 1A).

**FIGURE 2:**
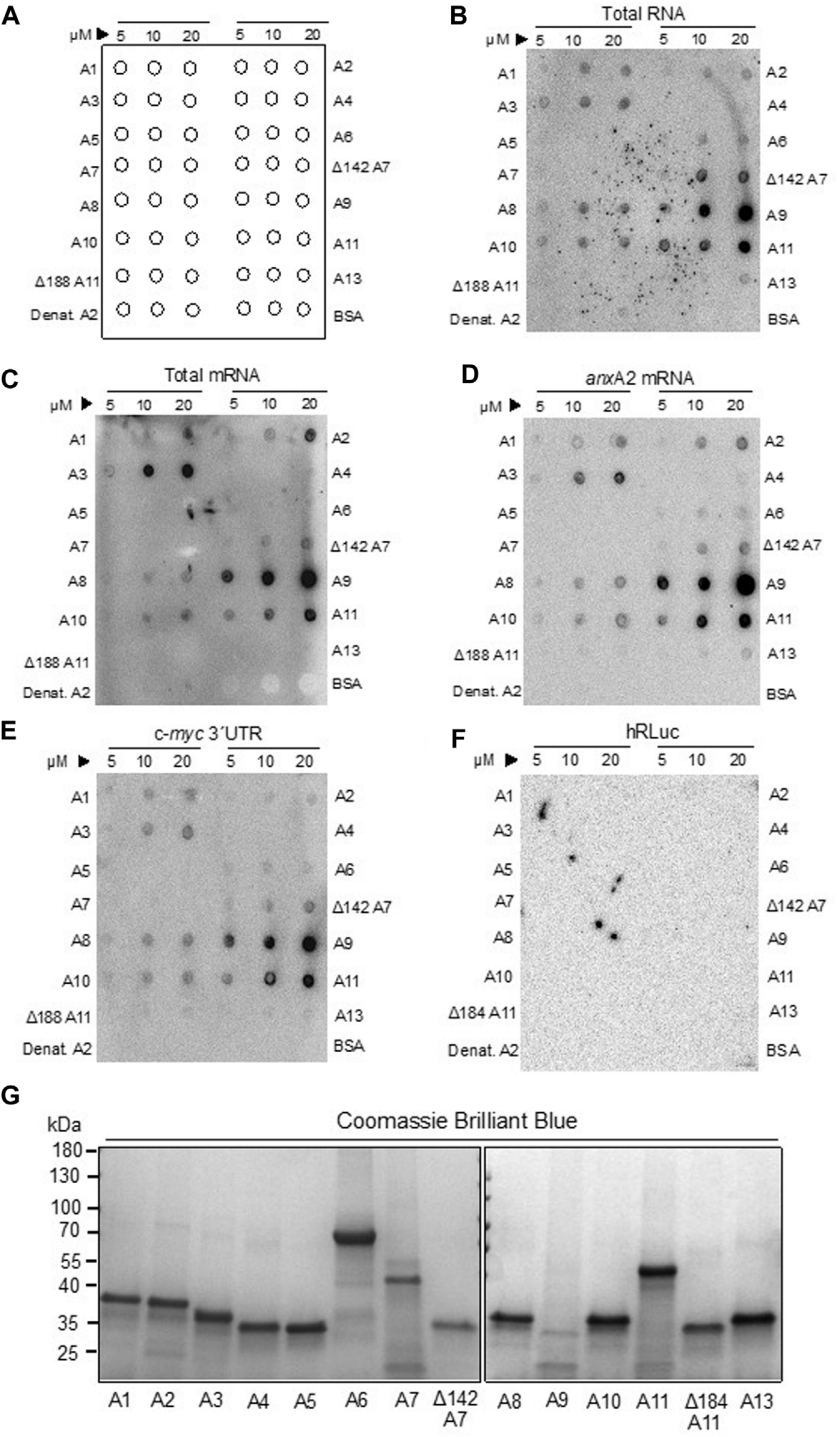
The binding of rat Anxs to total RNA, total poly(A)-containing mRNAs and specific RNAs. Panel **(A)** shows schematically the protocol for the spotting of proteins on membranes to ease the evaluation of the data. Recombinant rat AnxA1-to-AnxA11, AnxA13 and BSA were spotted on nitrocellulose membranes in 3 μL samples containing 5, 10 or 20 μM protein, as indicated in Panel **(A)**. Subsequently, the membranes were incubated for 30 min with 200,000 cpm of the following probes: [^32^P]-labeled total RNA [Panel **(B)**] or [^32^P]-labeled poly(A) -containing total mRNA [Panel **(C)**], *in vitro* transcribed [^32^P] rUTP-labeled anxA2 mRNA Panel **(D)**, c-myc 3′UTR [Panel **(E)**], or the coding region of hRLuc Panel **(F)**]. All incubations were carried out in RNA-binding buffer in the presence of 100 μM Ca^2+^ and 1 μg/μL tRNA to block unspecific binding of RNA. Heat-denatured AnxA2 and BSA were spotted as negative controls since they do not bind RNA. The radioactive signals were detected after 48 h exposure of the spot blots to a radiosensitive screen in a phosphor-imager (Fuji Bas-5000). The dark signals (spots) on the membrane indicate RNA binding to the indicated recombinant rat Anx proteins. Panel **(G)** Samples of Anxs as indicated were subjected to 4%–15% SDS-PAGE and the proteins were stained with Coomassie Brilliant Blue. Standard proteins (PageRuler prestained protein ladder) are indicated to the left. The spot blots are representative blots from 3 experiments.

We have previously found AnxA2 in translationally silent mRNP complexes obtained from the cytoskeletal fraction (Aukrust et al., 2017) and were therefore interested in investigating if other Anxs would also be present in these non-polysomal RNA granules/mRNP complexes. Thus, the cells were treated with cycloheximide (CHX) before harvest to stabilize the mRNAs already present in polysomes, which were then separated from non-polysomal mRNP complexes by ultracentrifugation. Subsequently, translationally silent non-polysomal mRNP complexes were captured from the post-polysomal supernatant above the sucrose cushion by oligo (dT) pull-down (Figure 1B). In addition to the previously demonstrated presence of AnxA2 in these mRNP complexes, AnxA1, AnxA4, AnxA5, AnxA6, AnxA7, AnxA10, AnxA11 and AnxA13 were also detected in the RNA granules (Figure 1B). RNase treatment abolished their binding to the oligo (dT) beads, indicating that the pull-down of the Anxs, like that of AnxA2, is RNA dependent (Figure 1B). We were particularly interested in the possible association of AnxA7, AnxA11 and AnxA13 with RNA since they are the oldest members of the mammalian Anx family (Morgan et al., 2004). Furthermore, AnxA1 is the closest relative of AnxA2 (Gerke and Moss, 2002) and thus, besides the latter, would be the most likely candidate to bind RNA. AnxA4, AnxA5 and AnxA6 appear to bind the specific transcripts with low affinity (Tables 2, 3). When competing with the other Anxs for binding to the specific RNA ligands (3′UTRs of anxA2 and c-myc mRNAs), AnxA13 appears to bind with low affinity (Figure 2) but bound with a K_D_ of about 250 nM when assayed alone. Its presence in mRNP complexes derived from PC12 cells shows that it is associated with RNA (Figure 1). We were interested in if both Anx modules of AnxA6 would bind RNA and therefore AnxA10 in addition to AnxA5 was included in the screening of the presence of Anxs in the mRNP complexes. Namely, AnxA6 was formed via chromosomal duplication during the genome expansion in early chordates (Morgan et al., 1999).

**TABLE 2:** An overview of the binding of rat Anxs to the different RNA probes as determined by spot blot and UV crosslinking (xlinking) experiments. xxx indicates strong binding and X indicates weak RNA binding with XX indicating intermediate binding. Also the presence of Anxs in mRNP complexes derived from PC12 cells detected with specific antibodies are indicated.

Protein	Total RNA	Total mRNA	anxA2	anxA2	c-myc	c-myc	Found in mRNP complexes
	Spot blot		mRNA	3′UTR	3′UTR	3′UTR	
			Spot blot	UV-xlinking	Spot blot	UV-xlinking	
AnxA1	xx	xx	xx	x	xx	x	Yes
AnxA2	xx	xxx	xx	xxx	x	xxx	Yes
AnxA3	xx	xxx	xxx	xxx	xx	xxx	
AnxA4	(x)		x	(x)		(x)	Yes
AnxA5				xx		(x)	Yes
AnxA6	X	x	x	x	(x)	xx	Yes
AnxA7				xxx		xxx	Yes
Δ142AnxA7	xxx	xx	xx	xx	x	(x)	
AnxA8	xx	xx	xx	xx	xx	xxx	
AnxA9	xxx	xxx	xxx	n.d	xxx	xxx	
AnxA10	xx	xx	xx	xxx	xx	xxx	Yes
AnxA11	xxx	xxx	xxx	xx	xxx	xxx	Yes
Δ188AnxA11			x	xx	x	xxx	
AnxA13	x		x	x	x	x	Yes

**TABLE 3:** Affinity and kinetic rate constants of various Annexin proteins for their interactions with untranslated regions of anxA2 or c-myc mRNAs.

RNA ID	Anxs	K_D_ (nM)	K_D_ Error (nM)	k_on_ (1/Ms) k	k_on_ Error	k_dis_(1/s)	k_dis_ Error	Full X^∧^2 F	Full R^∧^2
c-myc 3′UTR	AnxA2	73.9	5.19	1.26E+05	8.40E+03	9.28E-03	1.99E-04	0.053	0.92
	AnxA4	No binding							
	Δ142AnxA7	126	8.66	3.65E+03	1.77E+02	4.61E-04	2.24E-05	0.001	0.99
	Δ188AnxA11	124	47.9	1.06E+06	3.91E+05	1.31E-01	1.49E-02	0.005	0.93
	AnxA13	130	25.7	4.85E+05	9.15E+04	6.29E-02	3.79E-03	0.006	0.92
anxA2 3′UTR	AnxA2	192	11.2	6.41E+04	3.51E+03	1.23E-02	2.42E-04	0.063	0.95
	AnxA4	No binding							
	Δ142AnxA7	174	8.74	7.19E+03	2.95E+02	1.25E-03	3.65E-05	0.002	0.97
	Δ188AnxA11	>500	>500	2.00E+03	2.23E+04	3.87E-02	2.60E-03	0.003	0.90
	AnxA13	246	33.1	2.18E+05	2.77E+04	5.36E-02	2.46E-03	0.006	0.95
c-myc 5′UTR	AnxA2	37.2	3.81	2.46E+05	2.44E+04	9.17E-03	2.28E-04	0.013	0.90
	AnxA4	No binding							
	Δ142AnxA7	No binding							
	Δ188AnxA11	No binding							
	AnxA13	No binding							

### 3.2 Binding of Anxs to total RNA, total mRNA and specific RNAs as assessed by spot blots

Several Anxs associate in an RNase-sensitive manner with mRNP complexes captured by oligo (dT) magnetic beads (Figure 1B). However, besides showing the presence of these Anxs in mRNP complexes, these experiments do not clarify unambiguously whether they interact directly with RNA, or indirectly via other RNA-binding proteins. Thus, all the rat Anxs were cloned by RT-PCR—except the AnxA9 clone (the longest isoform), which was purchased—followed by subcloning, expression and purification. Based on the circular dichroism scanning spectra obtained (not shown), all Anx family members showed an overall α-helical content, except wt AnxA7 and wt AnxA11, which possess long unstructured N-termini. Since these two Anxs display a high tendency for aggregation and degradation, we also cloned and purified their N-terminally truncated versions, which are more stable and soluble (Lillebostad et al., 2020). The purified Anxs were spotted in their native form onto nitrocellulose membranes, with the three spots containing increasing amounts of protein (5, 10 and 20 μM) in a total volume of 3 μL (see Figure 2A, showing an outline of the spotted proteins). The membranes were then incubated with metabolically labeled total RNA and total poly(A)-containing mRNAs from PC12 cells (Figures 2B, C, respectively). Total poly(A)-containing mRNAs constitute about 2%–5% of total RNA. These experiments indicated that AnxA1, AnxA2 and AnxA3, as well as the core structures of AnxA7, AnxA8, AnxA9, AnxA10 and wt AnxA11 all bind total RNA and total poly(A)-containing mRNAs, whereas AnxA6 and AnxA13 only bind total RNA, but not total poly(A)-containing mRNAs (Figures 2B, C; Table 2). The differences in binding could possibly be explained if the latter group of Anxs bind regulatory non-coding RNAs (Monastyrskaya, 2018) or low abundance mRNAs. As AnxA6 and AnxA13 bind anxA2 mRNA and c-myc 3′UTRs (Figures 2D, E), the observed differences in binding are probably based on different specific activities of the two probes since total RNA has a lower specific activity due to short labeling time. However, it has been reported that some Anxs bind to non-coding RNAs (Monastyrskaya, 2018).

Next, we assayed the binding of all Anxs to full-length rat anxA2 mRNA (Figure 2D), which we have previously shown to interact with its cognate protein. In addition to the Anxs showing binding to total poly(A)-containing mRNAs, also AnxA4, AnxA6, Δ188AnxA11 and AnxA13 bound to the anxA2 mRNA although the signals were weak. Since we have previously shown that AnxA2 binds to the localization signal in the c-myc 3′UTR (Mickleburgh et al., 2005), it was also of interest to investigate whether the Anxs that bind to the anxA2 mRNA would show the same preference to the c-myc 3′UTR (Figure 2E). Also, it was of interest to detect possible redundancies. Indeed, the same Anxs, with the exception of AnxA4, appear to bind to this regulatory region of c-myc mRNA (Figure 2E).

Since in this experimental set-up all Anxs are spotted on the same membrane and therefore compete for the same RNA, their relative different affinities for RNA are also revealed to a certain degree. This depends, however, on the proportion of degraded and/or denatured protein in each spot since all steps before drying of the membranes and exposure to screens include several overnight incubations. It is also possible that those Anxs, which bind RNA with low affinity will not be readily revealed due to the lack of UV-crosslinking and thorough washing.

Finally, the binding of the Anxs to the coding region of Renilla luciferase mRNA was additionally investigated as a negative control (Figure 2F) since previous UV-crosslinking experiments showed that AnxA2 does not bind to this mRNA (Strand et al., 2021). Thus, the collective results from these experiments suggest that most Anxs, except possibly AnxA5, bind to mRNAs. Neither BSA nor heat-denatured AnxA2 bind RNA (Figure 2, B-F, lower row). Since we have previously shown that post-translational modifications of AnxA2 regulate its association with mRNA (Aukrust et al., 2017; Grindheim et al., 2017), it is likely that post-translational modifications also govern the association of these other Anxs with mRNA, being perhaps related to the affinity or specificity of interactions. When all Anx proteins were examined for their integrity by SDS-PAGE, it could be noted that AnxA9 is prone to aggregation and degradation (Figure 2G). In addition to the main monomeric form of the Anxs, dimeric forms of the Anxs are also visible as faint bands when all the proteins were analyzed by SDS-PAGE (Figure 2G).

### 3.3 Binding of Anxs to the 3′UTRs of two specific RNAs by UV-crosslinking

**FIGURE 3:**
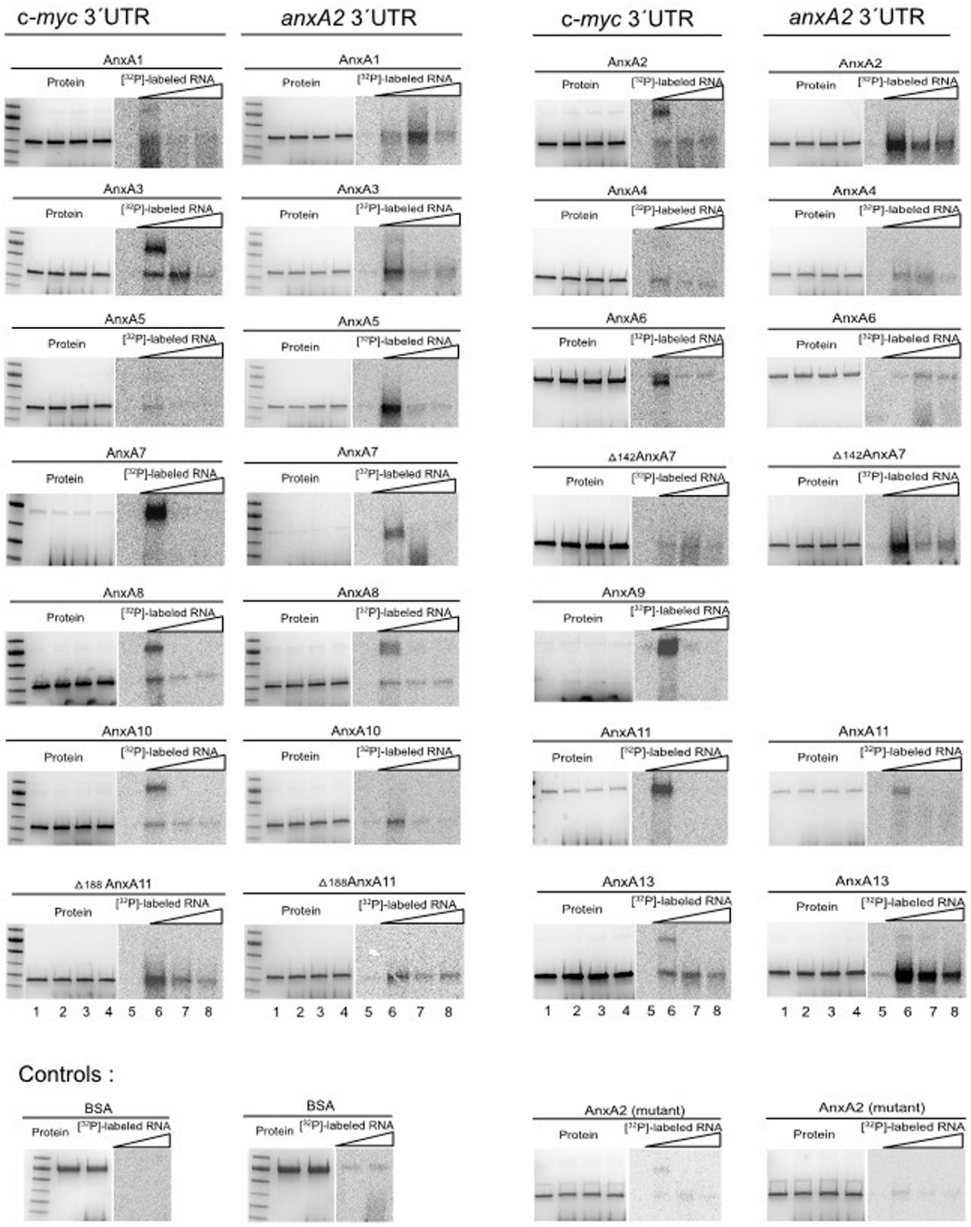
UV-crosslinking competition experiments employing rat Anxs and radiolabeled [α^32^P]-rUTP c-myc or anxA2 3′UTRs. 2 μM purified rat Anxs (except 0.4 µM of AnxA7; 0.3 µM of AnxA9 and 0.5 µM of AnxA11) were UV-crosslinked in the absence of RNA (lanes 1 and 5). 100,000 cpm of radiolabeled anxA2 3′UTR (7 fmoles) or c-myc 3′UTR (6 fmoles) were UV-crosslinked to purified rat Anxs in the absence (lanes 2 and 6) or presence of 25x (lanes 3 and 7), or 50x (lanes 4 and 8) molar excess of the corresponding unlabeled transcript. 2 μM BSA and mutant AnxA2 served as negative controls. After UV-crosslinking and RNase treatment, the samples were subjected to 4%–15% SDS-PAGE and the proteins were stained with Coomassie Brilliant Blue (lanes 1–4), whereafter the gels were dried. The [α^32^P]-rUTP-labeled RNA covalently bound to the respective Anxs, as indicated, was visualized using screens and phosphor-imaging following an overnight (c-myc 3′UTR) or 6 h (anxA2 3′UTR) exposure (lanes 5–8). PageRuler prestained protein ladder (from top to bottom: 100 kDa, 70 kDa (the most prominent band), 55 kDa, 40 kDa, 35 kDa and 25 kDa) are shown to the left of the AnxA1, AnxA3, AnxA5, AnxA7, AnxA8, AnxA10, Δ188AnxA11 and BSA samples. The representative images are from two experiments performed with competition while RNA-Anx binding was performed four times without competition.

Another means to study the interaction between RNA and protein is UV-crosslinking. This method is based on the formation of covalent bonds between proteins and RNA if they are in direct contact within a distance of about 1 Å during exposure to UV light (Ule et al., 2005). By contrast, proteins are not readily crosslinked under the same conditions (Greenberg, 1979). This is particularly important to keep in mind when using this approach with live cells (Ule et al., 2005). We tested this method first in combination with subsequent oligo (dT) affinity-purification of mRNP complexes. However, the use of the original protocol led to variable protein degradation, affecting the Anxs more than certain other proteins, such as PABP1 (results not shown). We therefore adopted the *in vitro* UV-crosslinking method employing purified recombinant Anxs and purified *in vitro* transcribed (^32^P) rUTP-labeled RNA (Figure 3). In this approach, 2 µM of the Anxs was used (except 0.4 µM of AnxA7, 0.3 µM of AnxA9 and 0.5 µM of AnxA11) in combination with 100,000 cpm of transcript. This UV-crosslinking procedure requires a much shorter incubation time, thus resulting in reduced degradation of unstable proteins.

We performed UV-crosslinking experiments with all rat Anxs and the 3′UTRs of c-myc and anxA2 mRNAs as indicated (Figure 3). Binding to the radiolabeled 3′UTR transcripts (Figure 3, lanes 2 and 6) was competed by incubation with 25x (Figure 3, lanes 3 and 7) or 50x (Figure 3, lanes 4 and 8) molar excess of the corresponding unlabeled transcript. Previously, using the surface plasmon resonance technique, we obtained evidence that the binding of AnxA2 to RNA in the presence of Ca^2+^ induces a conformational change in the protein (and possibly also in the RNA). This leads to an extremely slow off rate (Aukrust et al., 2007) explaining why competition would decrease the signal but not abolish it completely. This may not necessarily be the situation for all Anxs. In the case of the c-myc 3′UTR, competition was effective for the monomeric forms of AnxA1, AnxA5, AnxA6, wt AnxA7, AnxA8, AnxA9, AnxA10, AnxA11 and Δ188AnxA11, and in the case of the dimeric forms of AnxA1, AnxA2, AnxA3, AnxA8, AnxA9, AnxA10 and AnxA13. Regarding the anxA2 3′UTR competition was effective for the monomeric forms of AnxA2, AnxA3, AnxA5, wt AnxA7, Δ142AnxA7 AnxA8, AnxA10, wt AnxA11, Δ188AnxA11 and partly AnxA13, while being effective for the dimeric forms of AnxA3 and AnxA8. To provide an overview, the results from the spot blot and the UV-crosslinking experiments are summarized in Table 2.

### 3.4 Determination of the K_D_ for the interaction between RNA and specific Anxs

To study in more detail the apparent dissociation constant K_D_ of the interactions, real-time binding assays of AnxA2, AnxA13 and the core structures of AnxA7 and AnxA11 with the c-myc or anxA2 3′UTRs were performed by biolayer interferometry (BLI). AnxA2, Δ142AnxA7 and Δ188AnxA11 were chosen as Anxs showing a known or an overall strong binding to RNA, while AnxA4 was chosen for its apparent low or lack of binding to RNA. AnxA13 was selected as an Anx with an apparent relatively intermediate binding to RNA (Table 2) and as the oldest member of the mammalian Anx family (Morgan et al., 2004). The core structures of AnxA7 and AnxA11 were used since they are more soluble and less prone to aggregation than the full-length proteins. The 3′-end biotinylated 3′UTRs were in the immobilized phase while the Anxs were in the soluble phase. The K_D_s (Table 3) were calculated from the kinetic data (Supplementary Figure S2) and are in the nM range (∼75–250 nM) for AnxA2, AnxA13 and the core structures of AnxA7 and AnxA11 for interaction with c-myc 3′- and anxA2 3′-UTRs. By contrast, the core structure of AnxA11 has a much lower affinity for anxA2 3′UTR (∼2 µM) indicating some selectivity. Only AnxA2 binds to the 5′UTR of c-myc mRNA indicating specificity most likely related to the regulation of translation. AnxA4 did not bind to the two specific 3′UTRs.

## 4 Discussion

### 4.1 Anxs are present in mRNP complexes derived from PC12 cells

Our previous studies revealed that AnxA2 is enriched in the cytoskeletal fraction where an intracellular pool of AnxA2 associates with a subpopulation of mRNAs associated with the cytoskeleton (Vedeler and Hollas, 2000). We also showed that this interaction is regulated by Ca^2+^ and several post-translational modifications (Vedeler et al., 2012; Grindheim et al., 2014; Grindheim et al., 2017). Furthermore, we have determined the RNA-binding site in domain IV of the core structure of AnxA2 (Aukrust et al., 2007), which–at least in structural terms—is similar for all members of the Anx family (Gerke and Moss, 2002). We therefore speculated that the RNA-binding ability could be a feature shared by most Anxs, if not all of them.

Only a subpopulation of AnxA2 is associated with RNA and it has been suggested that this fraction corresponds to about 10% of the total protein (Arrigo et al., 1983). These are rough estimates that are expected to depend on the cell type and the physiological status of the cells. Similar estimates are likely to apply for at least some of the other Anxs as well.

AnxA2 has previously been found in microtubule-bound mRNP complexes associated with the MIDI protein, which is related to the Opitz BBB/G syndrome as well as in mRNP complexes containing the collagen prolyl 4-hydroxylase-α(I) mRNA (Fahling et al., 2006; Aranda-Orgilles et al., 2008). In PC12 cells, AnxA2 was also shown to be present in mRNP complexes containing the anxA2 and c-myc mRNAs (Vedeler and Hollas, 2000; Hollas et al., 2006; Vedeler et al., 2012). It also associates with c-myc mRNA in HeLa cells (Filipenko et al., 2004). AnxA10 is structurally a typical member of the Anx family: however, not functionally and appears to be specifically involved in paraspeckle-associated mRNA regulation or processing (Quiskamp et al., 2014). In a proteomic screen, AnxA7 and Anx11 have been detected in mRNP complexes from human embryonic kidney cells (Baltz et al., 2012), and AnxA11 is a component of mRNP complexes from U2OS bone osteosarcoma cells (Liao et al., 2019). Using “interactome capture”, an approach relying on the capture of poly(A)-binding proteins and stabilizing their interaction, AnxA1, AnxA2, AnxA7 and AnxA11 were identified in HeLa cells (Castello et al., 2012), further supporting our present results. Since AnxA7, AnxA11 and AnxA13 are the oldest mammalian Anxs (Iglesias et al., 2002; Morgan et al., 2004), it is tempting to suggest that the RNA-binding property of this protein family could be an ancient trait. The analysis of polysomes derived from the cytoskeletal fraction using specific antibodies (Figure 1; Supplementary Figure S1) against, AnxA1, AnxA2, AnxA4, AnxA5, AnxA6, AnxA7, AnxA10, AnxA11 and AnxA13 indicated that only a minor fraction of the proteins can be found in the polysomal fraction.

This was most evident for AnxA2 and AnxA13 (Figure 1A). AnxA1, AnxA2, AnxA4, AnxA5, AnxA6, AnxA7, AnxA10, AnxA11 and AnxA13 were all identified in mRNP complexes in the post-polysomal cytoskeletal fraction after oligo d(T) pulldown and Western blot analysis (Figure 1B). These results support the conclusion that—besides AnxA2—other Anxs most likely also associate with mRNAs. Their association with mRNP complexes is RNA-dependent as it is destroyed by RNase treatment (Figure 1B). It should be noted that the presence of Anxs in mRNP complexes derived from total lysates was not as clearcut (results not shown). This could be due to the presence of detergents in the lysates, but most likely reflects the fact that the Anxs bind to a subpopulation of mRNAs associated with the cytoskeleton fraction. Recently, we also observed the presence of AnxA2 in cap pull down mRNP complexes from the cytoskeletal fraction, but not from total lysates (Grindheim et al., 2023).

What is the function of the Anxs in mRNP complexes? Of interest, regarding AnxA2, it has been reported to associate with poly(A) binding protein 1 (PABP1) (Filipenko et al., 2004; Grindheim et al., 2023). Furthermore, AnxA2 binds to the localization signal in the c-myc 3′UTR (Mickleburgh et al., 2005). It is possible that by interacting with PABP1, AnxA2 competes with eIF4G for binding to the cap-binding protein eIF4E (Kats and Klann, 2019). Alternatively, it may bind directly to eIF4E (Grindheim et al., 2023) and thereby inhibit translation, while the RNA granule/mRNP complex is undergoing transport. Studies of AnxA11 open the general possibility that Anxs simultaneously tether RNA granules to vesicles (Liao et al., 2019), thereby rendering intracellular transport more energy efficient.

### 4.2 *In vitro* binding of Anxs to different RNA transcripts

It appears that many of the Anxs preferentially bind the RNA transcripts as a dimer (Figure 3). Indeed, AnxA1 (Pepinsky et al., 1989), AnxA2 (López-Rodríguez et al., 2018) and AnxA13 (McCulloch et al., 2019) have previously been reported to form dimers. Thus, in the case of AnxA1, AnxA2, AnxA3, AnxA8, AnxA9, AnxA10 and AnxA13 it is the dimeric form of the protein that mainly binds RNA, although only a faint band is visible in the Coomassie Brilliant Blue stained gels (Figures 2G, 3, lanes 1–4). Other RNA-binding proteins have been shown to bind RNA in their monomeric form while their dimerization is necessary for strong RNA binding (Piron et al., 1999). Furthermore, oligomeric forms of Anxs are known to occur naturally [see (Lillebostad et al., 2020)] and oligomerization could be induced when binding to certain ligands. It should be noted that all Anxs, except AnxA7, AnxA9 and AnxA13 (since their concentrations were very low), were gel filtrated. It is possible that β-mercapto ethanol may not give complete reduction of all disulfide bonds (Valetti and Sitia, 1994) and thus the Anx dimers detected by SDS-PAGE could remain linked via disulfide bridges. However, it has also been reported that proteins may form tight noncovalent interactions (Lüders et al., 2003). At present we do not know the nature of the Anx dimers. Interestingly, however, the RNA-binding sites in domain IV of the Ca^2+^-bound dimer of AnxA2 are exposed at the side of the structure that is opposite to the site of dimerization (pdb: 1XJL) (Rosengarth and Luecke, 2004).

UV-crosslinking of RNA to the Anxs does not lead to detectable protein retardation during 4%–15% SDS-PAGE, indicating the crosslinking of a maximum of 4–6 RNase-resistant nucleotides (Figure 3, compare lane 1 with lanes 2–4). Taking into account the signal intensities and the competition between radiolabeled and unlabeled RNAs, it appears that AnxA1, AnxA6, AnxA7, AnxA11 and Δ188AnxA11—in particular the dimers of AnxA2, AnxA3, AnxA8, AnxA9 and AnxA10, and only the dimer of AnxA13—bind with higher affinity to the c-myc 3′UTR than do AnxA4, AnxA5, Δ142AnxA7 and the mutant form of AnxA2 which does not bind RNA (Aukrust et al., 2007; Solbak et al., 2017). Using the same criteria, AnxA2, AnxA3, AnxA5, AnxA7, Δ142AnxA7, AnxA8 (particularly the dimer), AnxA10, AnxA11, Δ188AnxA11, and AnxA13 seem to bind with higher affinity to the anxA2 3′UTR, as compared to AnxA1, AnxA4, AnxA6 and the mutant AnxA2.

In addition to UV-crosslinking experiments, we also used binding assays involving spotting of the native proteins in three concentrations onto the same membrane (immobilized phase) for incubation with the radio-labeled transcript in solution (Figure 2). Thus, the different Anxs compete for binding, and it is possible that Anxs with low RNA-binding affinity may not compete successfully. However, overall, the different experimental approaches are generally in agreement.

The K_D_s of selected Anxs were determined using the real-time BLI. Biotinylated c-myc 5′UTR, c-myc or anxA2 3′UTRs were immobilized onto the biosensor surface while the Anxs (the analyte) were kept in solution. All the selected Anxs (AnxA2, Δ142AnxA7, Δ188AnxA11 and Anx13), except AnxA4, interacted with the c-myc and anxA2 3′UTRs with K_D_s in the nM range. Regarding the interaction between Δ188AnxA11 and anxA2 3′UTR the K_D_ was found to lie in the µM range (Table 3) indicating a lower affinity. Previous studies using surface plasmon resonance (SPR) have shown that AnxA2 interacts with an 8-mer RNA (5′-GGGGAUUG) with a K_D_ of about 10 nM (Solbak et al., 2017). Furthermore, AnxA2 binds to poly(G) with a K_D_ of about 60 nM (Filipenko et al., 2004).

Overall, the data from the three types of experiments used in this study are in accordance with each other. Based on our spot blot (Figure 2), UV-crosslinking (Figure 3) and BLI experiments AnxA4 does not appear to bind (or binds very weakly) to the employed transcripts (Table 3). However, we cannot rule out that AnxA4 may bind other RNA transcripts since we observed that it binds to metabolically radiolabeled total mRNAs in the presence of 1 mM calcium (data not shown), which is not physiologically relevant. In addition, AnxA4 is present in mRNP complexes obtained from the cytoskeletal fraction of PC12 cells in the presence of 100 µM calcium (Figure 1B). This discrepancy could be related to the types of mRNAs it interacts with, as well as post-translational modifications.

AnxA9 most likely became degraded in the course of these experiments. It is notoriously a very difficult protein to study as it aggregates easily and will only remain intact for a few hours on ice. Also, it does not tolerate multiple rounds of freezing and thawing and appears to become degraded by UV-crosslinking. Thus, it was very difficult to obtain reliable data for this particular Anx. However, it evidently binds the c-myc 3′UTR (Figure 3). Generally, it was observed that when the Anx proteins denature, they lose their ability to bind RNA, leading to wrong conclusions regarding their RNA binding properties. In this study, we have optimized the production of recombinant Anxs. However, full-length AnxA7 and full-length AnxA11, as well as AnxA9, were notoriously problematic proteins.

Regarding the binding of the Anxs to the 3′-regulatory regions of c-myc and anxA2 mRNAs, the most striking differences were that the core structure of AnxA7, as well as AnxA5 and AnxA13 bind strongly to the anxA2 3′UTR (Figures 2, 3 as well as Table 3). As pointed out earlier, AnxA7 and AnxA13 belong to the oldest members of the Anx family (Moss and Morgan, 2004). Furthermore, it was of interest to investigate whether both core structures of AnxA7 and AnxA11 would be involved in the interaction with mRNA, as shown in the case of AnxA2 (Aukrust et al., 2007). In addition, a recent study proposed that the long and mostly unstructured N-terminus of AnxA11 is involved in its binding to mRNA, while the core structure is involved in lipid binding to mediate long-distance co-transport of RNA granules (mRNP complexes) with lysosomes (Liao et al., 2019). Here we show that the core structures of AnxA7 and AnxA11 both have the capacity to bind RNA (Figures 2, 3 as well as in particular Table 3). However, this does not rule out that a second RNA binding site is present in their N-terminus. If the RNA-binding site of AnxA11 is similar to that of AnxA2, it is expected to be located at the convex side facing the long N-terminus, based on analysis of its crystal structure (Lillebostad et al., 2020). It is possible that full-length AnxA7 and AnxA11 need to interact with specific protein ligands to expose their RNA-binding site(s) in the core structure.

It was surprising to observe that most of the Anxs bind the anxA2 and c-myc 3′UTRs. This could indicate redundancy or compensatory effects related to RNA-binding amongst the Anxs in line with the notion that AnxA7 is upregulated when AnxA2 is knocked-down in PC12 cells by a potent Antisense RNA (Aareskjold et al., 2019). This suggests that the Anxs are very important multifunctioning proteins with backing from their family members, at least regarding some functions but maybe not all (Grewal et al., 2016). Only AnxA2 and not AnxA4, AnxA13 nor the core structures of AnxA7 and AnxA11 bind to the c-myc 5′UTR indicating some specificity. Collectively, our data show that the interaction of Anxs with mRNA is direct and that most if not all Anxs bind RNA. It appears likely that the different Anxs bind a specific sub-set of mRNAs transported to cytoskeleton-bound polysomes with partial redundancy.

### 4.3 Alignment of the RNA-binding site in domain IV of AnxA2 with the corresponding region of the other rat Anxs

The RNA-binding site present in AnxA2 resides in helices C-D of domain IV, the last domain of its C-terminal core structure (Aukrust et al., 2007). Analysis of AnxA2 where the different amino acid residues thought to participate in RNA recognition (Lys308-Lys310, Lys313, Tyr317 and Gln321—discussed in (Aukrust et al., 2007) - were mutated to Ser confirmed the predominant involvement of six surface-exposed residues in domain IV in RNA binding. The residues Lys308-Lys310 and Lys313, as well as Tyr317 and Gln321 are involved in RNA recognition, since when all six residues were mutated, the RNA binding capability of the ensuing AnxA2 variant was inhibited by approximately 98% (Aukrust et al., 2007; Solbak et al., 2017). Mutation of the Lys308-Lys310 residues resulted in 90% inhibition in RNA binding. In AnxA2, Lys308-Lys310 resides in helix C, Lys313 in the loop, while Tyr317 and Gln321 are present in helix D. All these residues are highly exposed. Clusters of basic amino acid residues have been shown to contribute to RNA binding (Houmani and Ruf, 2009). The positively charged residues most likely bind to the negatively charged phosphate backbone of RNA by electrostatic interactions, which is a common type of interaction between RNA and RNA-binding proteins (Law et al., 2006). The interaction between AnxA2 and its cognate mRNA as studied by SPR appears to be complex and sequential consisting of an initial fast phase of recognition dominated by electrostatic interactions, most likely between positively charged lysine residues and the phosphate backbone of RNA, followed by a second phase contributing to the specificity of the interaction (Aukrust et al., 2007).

**FIGURE 4:**
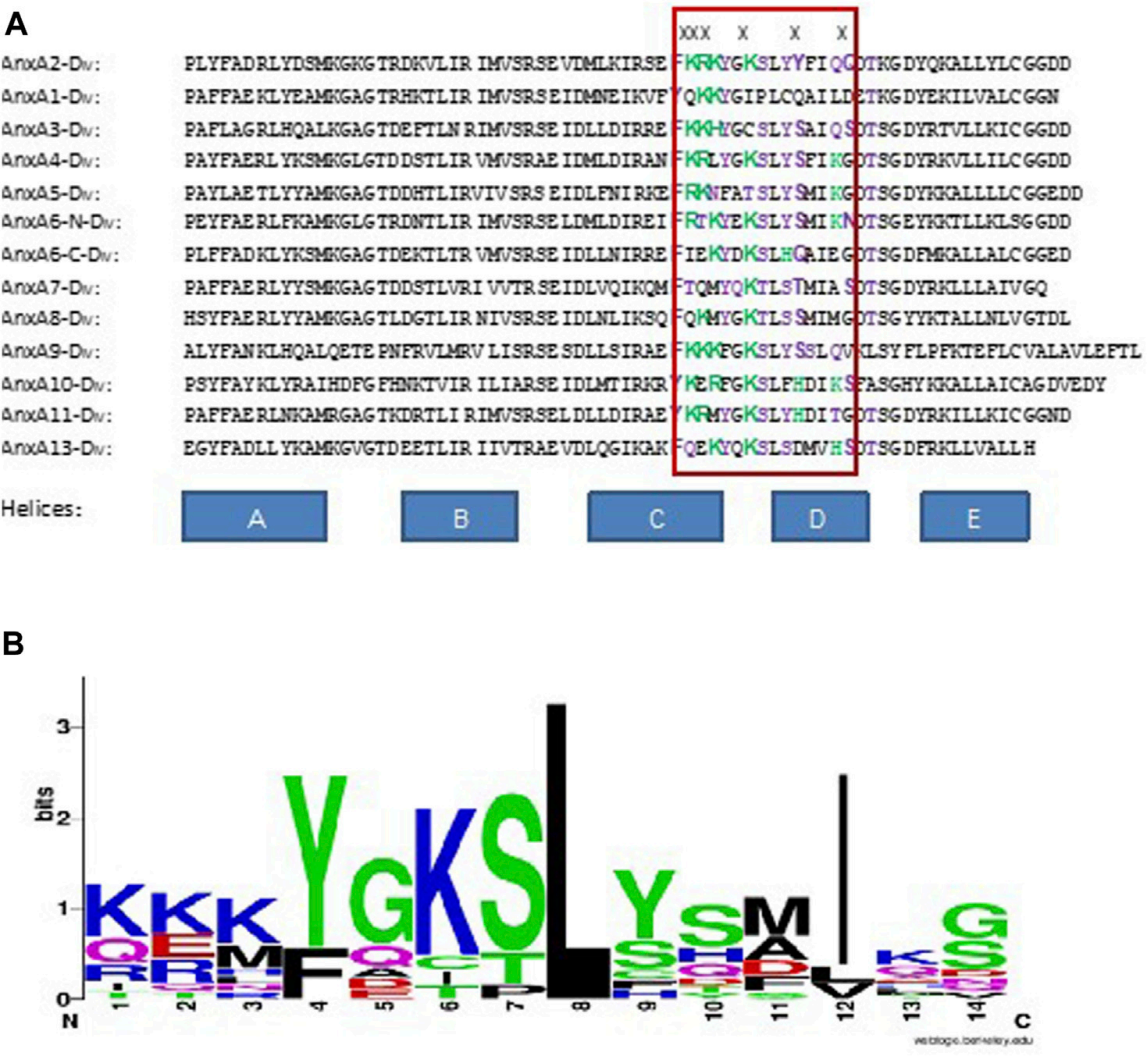
Alignment of the RNA-binding site in domain IV of AnxA2 (indicated by the red box) with corresponding sites in the other rat Anxs. **(A)** In the “RNA-binding site”, positively charged amino acid residues are labeled green and polar residues purple. X above the sequences indicates the amino acid residues involved in RNA-binding in AnxA2. **(B)** WebLogo representation (Schneider and Stephens, 1990; Crooks et al., 2004) of the alignment of the RNA-binding site in AnxA2 shown inside the red box in Panel A. The overall height of a stack indicates the sequence conservation at the position of that amino acid residue among the 13 different sequences, while the height of symbols within the stack indicates the relative frequencies of the various amino acid residues found at that position in the RNA-binding site.

Alignment of the sequences of all rat Anxs shows that AnxA2, AnxA3 and AnxA9 have three positively charged amino acid residues at equivalent sites as compared to the positions 308–310 in AnxA2. AnxA9 and AnxA2, which are closely related (Moss and Morgan, 2004), have residues with the same chemical properties (regarding the side chains) in all six residues believed to reside in the RNA binding site. The other RNA-binding Anxs, except AnxA7, have 1-2 positively charged amino acid residues in the sites corresponding to positions 308–310 in AnxA2. AnxA7 stands out from the rest in having no positively charged amino acids at these positions (Figure 4A). While the alignment of the RNA-binding rat Anxs does not reveal a conserved consensus sequence involved in mRNA binding, it indicates that positively charged residues play a key role in the binding, since several of the RNA-binding Anxs contain such residues at these positions (compare Figures 2, 3; Table 3 with Figure 4). Furthermore, the sites in the other Anxs, corresponding to the AnxA2 RNA-binding site, are rich in positively charged and polar amino acid residues (Figures 4A, B).

The number of positively charged amino acids present at the sites equivalent to positions 308–310 in AnxA2, however, does not seem to impact the binding affinity of the particular Anx. Accordingly, AnxA3 with three positively charged amino acids does not bind with apparently higher affinity to mRNA than AnxA1 or AnxA8 containing two and one positively charged amino acids, respectively. Producing crystals of Anxs with bound RNA has turned out to be a difficult task (work in progress).

Thus, while a single RNA-binding site has been revealed in AnxA2 (Aukrust et al., 2007), it is possible that other Anxs may harbor multiple RNA-binding sites. AnxA7, AnxA11 and AnxA13 are the oldest members of the AnxA family, with orthologs discovered in other species (Iglesias et al., 2002; Moss and Morgan, 2004). Notably, AnxA7, AnxA11 and AnxA13 all bind RNA (Table 2). Thus, it will be very interesting to find out whether invertebrate Anxs also bind RNA. We performed a phylogenetic relationship and multisequence alignment of domain IV of various invertebrate and vertebrate Anxs (Supplementary Figures S3, S4) and noticed that the AnxA2 type RNA-binding site is absent in Anxs from bacteria, protozoans and slime molds, but present in Anxs of some plants, fungi and algae Anxs (Supplementary Figure S4). However, the consensus sequence of this multiple alignment is KKKYG (+DFPL) KSLY (Supplementary Figure S5) with the amino acids in the parenthesis being extra and thus different from the equivalent site in all mammalian Anxs suggesting that the first three positively charged amino acids are relatively conserved (Supplementary Figure S3). These conclusions may have to be revised when more Anxs are discovered.

An attractive functional implication of our present findings is that Anxs play a role in the coordinated transport of vesicles and mRNAs to specific subcellular locations to co-regulate cellular responses to specific signals. Thus, multiple Anxs may tether the mRNAs to vesicles as first shown for AnxA11 (Liao et al., 2019). Recent studies of the fungus, Ustilago maydis, showed that endosomal Upa1 couples mRNA and vesicle transport by binding to both RNA and the vesicle membrane, supporting the idea that endosomal proteins link these processes (Pohlmann et al., 2015).

In conclusion, - using different experimental approaches we show - that the direct RNA-binding property–first discovered and characterized in the case of AnxA2 (Arrigo et al., 1983; Vedeler and Hollas, 2000; Filipenko et al., 2004)—appears to be shared by most Anxs. Thus, it appears to represent an ancient mammalian trait of this protein family.

## Data Availability

The original contributions presented in the study are included in the article/Supplementary Material, further inquiries can be directed to the corresponding author.

## Supplementary material

Supplementary Figure S1 Specificities of Anx antibodies.

Supplementary Figure S2
**BLI analysis of Anxs-RNA interactions.** The sequences of various RNAs are described in Section 2.6 and kinetic parameters are detailed in Table 1. Binding was monitored on the OctetRED96 instrument (FortéBio, Menlo Park, USA) by allowing the association for 180 sec and the dissociation for 200 sec. RNA analytes are mentioned on the top, whereas sensorgrams of individual Anx partners are labeled. In each sensorgram, fitting is shown in red.

Supplementary Figure S3
**Phylogenetic relationship of domain IV of various Invertebrate and vertebrate Anxs**. Sequences of Annexin domain IV from Human, Rat, Mouse, Bovine, Arabidopsis thaliana (ARATH, Plantae), Oryza sativa (ORYSJ, Plantae), Dunaliella tertiolecta (DUNTE, Algae), Phaeocystis antarctica (PHAN, Algae), Coccolithus braarudii (COBRA, Algae), Phaeocystis antarctica (PHAN, Algae), Klebsormidium nitens (KLEN, Algae), archaeon (ARCH, Archaea), Penicillium flavigenum (PENFL, Fungi), Albugo candida (Aca, Fungi), Aspergillus Candida (ASPCN, Fungi), Polysphondylium pallidum (POLPP, slime mold), Fonticula alba (FONAL, cellular slime mold), Flavobacterium alkalisoli (FLAO, Bacteria), Flavobacterium alkalisoli (FLAO, Bacteria), Thermus tengchongensis (thete, Bacteria), Nocardia seriolae (NoCa, Bacteria), Palpitomonas bilix (PABIL, Amoeboid protists), Reticulo5myxa filosa (RETFI, amoeboid protists), Dictyostelium discoideum (DICDI, Amoeboid protists), Arcella intermedia (ARIN, Amoeboid protists), Spironucleus barkhanus (SPIBA, Protozoan), Thecamonas trahens (THETB, Protozoan) were aligned for phylogentic analysis using Neighbor-joining method. Branch lengths reflecting the extent of sequence divergence is shown. Separation of Protist Anxs from vertebrate and plant Anx is clearly visible.

Supplementary Figure S4
**MultiSequence Alignment of domain IV of various Invertebrate and vertebrate Anxs**. Sequences of Annexin domain IV from Human, Rat, Mouse, Bovine, Arabidopsis thaliana (ARATH, Plantae), Oryza sativa (ORYSJ, Plantae), Dunaliella tertiolecta (DUNTE, Algae), Phaeocystis antarctica (PHAN, Algae), Coccolithus braarudii (COBRA, Algae), Phaeocystis antarctica (PHAN, Algae), Klebsormidium nitens (KLEN, Algae), archaeon (ARCH, Archaea), Penicillium flavigenum (PENFL, Fungi), Albugo candida (Aca, Fungi), Aspergillus Candida (ASPCN, Fungi), Polysphondylium pallidum (POLPP, slime mold), Fonticula alba (FONAL, cellular slime mold), Flavobacterium alkalisoli (FLAO, Bacteria), Flavobacterium alkalisoli (FLAO, Bacteria), Thermus tengchongensis (thete, Bacteria), Nocardia seriolae (NoCa, Bacteria), Palpitomonas bilix (PABIL, Amoeboid protists), Reticulo5myxa filosa (RETFI, amoeboid protists), Dictyostelium discoideum (DICDI, Amoeboid protists), Arcella intermedia (ARIN, Amoeboid protists), Spironucleus barkhanus (SPIBA, Protozoan), Thecamonas trahens (THETB, Protozoan) were aligned using Clustal Omega.

Supplementary Figure S5
**Multiple sequence alignment of domain IV of various vertebrate Anxs**. Sequences of Anx domain IV from human, rat, mouse, and bovine species were aligned using Clustal Omega.
