# Supplementary material for: RNA-binding is an ancient trait of the Annexin family
Source: Front Cell Dev Biol. 2023 Jun 15;11:1161588. doi: 10.3389/fcell.2023.1161588 (PMC10311354; doi:10.3389/fcell.2023.1161588)
Supplement: Supplementary file 3 [file Presentation1.pdf]

Captions to Supplementary Figures:

Supplementary Figure S1

Specificities of Anx antibodies.

Supplementary Figure S2

BLI analysis of Anxs-RNA interactions. The sequences of various RNAs are described in Section 2.6 and kinetic parameters are detailed in Table 3. Binding was monitored on the OctetRED96 instrument (FortéBio, Menlo Park, USA) by allowing the association for 180 sec and the dissociation for 200 sec. RNA analytes are mentioned on the top, whereas sensorgrams of individual Anx partners are labeled. In each sensorgram, fitting is shown in red.

Supplementary Figure S3

Phylogenetic relationship of domain IV of various Invertebrate and vertebrate Anxs. Sequences of Annexin domain IV from Human, Rat, Mouse, Bovine, *Arabidopsis thaliana* (ARATH, Plantae), *Oryza sativa* (ORYSJ, Plantae), *Dunaliella tertiolecta* (DUNTE, Algae), *Phaeocystis antarctica* (PHAN, Algae), *Coccolithus braarudii* (COBRA, Algae), *Phaeocystis antarctica* (PHAN, Algae), *Klebsormidium nitens* (KLEN, Algae), *archaeon* (ARCH, Archaea), *Penicillium flavigenum* (PENFL, Fungi), *Albugo candida* (Aca, Fungi), *Aspergillus Candida* (ASPCN, Fungi), *Polysphondylium pallidum* (POLPP, slime mold), *Fonticula alba* (FONAL, cellular slime mold), *Flavobacterium alkalisoli* (FLAO, Bacteria), *Flavobacterium alkalisoli* (FLAO, Bacteria), *Thermus tengchongensis* (thete, Bacteria), *Nocardia seriolae* (NoCa, Bacteria), Palpitomonas bilix (PABIL, Amoeboid protists), *Reticulo5myxa filosa* (RETFI, amoeboid protists), *Dictyostelium discoideum* (DICDI, Amoeboid protists), *Arcella intermedia* (ARIN, Amoeboid protists*), Spironucleus barkhanus* (SPIBA, Protozoan), *Thecamonas trahens* (THETB, Protozoan) were aligned for phylogentic analysis using **Neighbor-joining** method. Branch lengths reflecting the extent of sequence divergence is shown. Separation of Protist Anxs from vertebrate and plant Anx is clearly visible.

Supplementary Figure S4

MultiSequence Alignment of domain IV of various Invertebrate and vertebrate Anxs. Sequences of Annexin domain IV from Human, Rat, Mouse, Bovine, *Arabidopsis thaliana* (ARATH, Plantae), *Oryza sativa* (ORYSJ, Plantae), *Dunaliella tertiolecta* (DUNTE, Algae), *Phaeocystis antarctica* (PHAN, Algae), *Coccolithus braarudii* (COBRA, Algae), *Phaeocystis antarctica* (PHAN, Algae), *Klebsormidium nitens* (KLEN, Algae), *archaeon* (ARCH, Archaea), *Penicillium flavigenum* (PENFL, Fungi), *Albugo candida* (Aca, Fungi), *Aspergillus Candida*

(ASPCN, Fungi), *Polysphondylium pallidum* (POLPP, slime mold), *Fonticula alba* (FONAL, cellular slime mold), *Flavobacterium alkalisoli* (FLAO, Bacteria), *Flavobacterium alkalisoli* (FLAO, Bacteria), *Thermus tengchongensis* (thete, Bacteria), *Nocardia seriolae* (NoCa, Bacteria), *Palpitomonas bilix* (PABIL, Amoeboid protists), *Reticulo5myxa filosa* (RETFI, amoeboid protists), *Dictyostelium discoideum* (DICDI, Amoeboid protists), *Arcella intermedia* (ARIN, Amoeboid protists), *Spironucleus barkhanus* (SPIBA, Protozoan), Thecamonas trahens (THETB, Protozoan) were aligned using Clustal Omega.

Supplementary Figure S5

Multiple sequence alignment of domain IV of various vertebrate Anxs. Sequences of Anx domain IV from human, rat, mouse, and bovine species were aligned using Clustal Omega.
