# Supplementary material for: RNA-binding is an ancient trait of the Annexin family
Source: Front Cell Dev Biol. 2023 Jun 15;11:1161588. doi: 10.3389/fcell.2023.1161588 (PMC10311354; doi:10.3389/fcell.2023.1161588)
Supplement: Supplementary file 6 [file Image2.pdf]

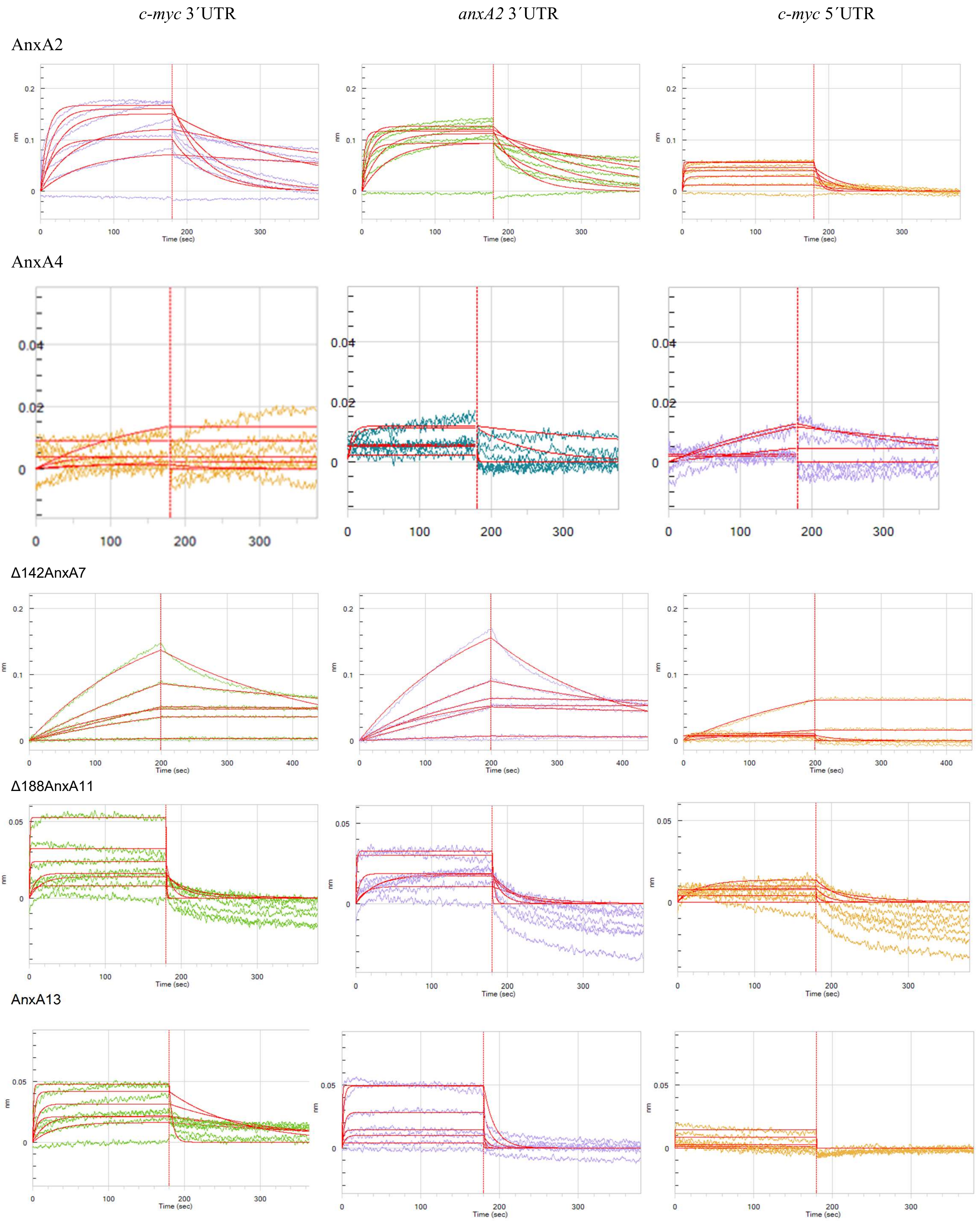

Supplementary Figure S2. BLI analysis of Anxs-RNA interactions. The sequences of various RNAs are described in Section 2.6 and kinetic parameters are detailed in Table 3. Binding was monitored on the OctetRED96 instrument (FortéBio, Menlo Park, USA) by

allowing the association for 180 sec and the dissociation for 200 sec. RNA analytes are mentioned on the top, whereas, sensorgrams of individual Anx partners are labeled. In each sensorgram, fitting is shown in red.
